# Novel Hybrid 1,2,4- and 1,2,3-Triazoles Targeting Mycobacterium Tuberculosis Enoyl Acyl Carrier Protein Reductase (InhA): Design, Synthesis, and Molecular Docking

**DOI:** 10.3390/ijms23094706

**Published:** 2022-04-24

**Authors:** Maged A. El Sawy, Maram M. Elshatanofy, Yeldez El Kilany, Kamal Kandeel, Bassma H. Elwakil, Mohamed Hagar, Mohamed Reda Aouad, Fawzia Faleh Albelwi, Nadjet Rezki, Mariusz Jaremko, El Sayed H. El Ashry

**Affiliations:** 1 Department of Pharmaceutical Chemistry, Faculty of Pharmacy, Pharos University, Alexandria 21311, Egypt; 2 Department of Chemistry, Faculty of Science, Alexandria University, Alexandria 21321, Egypt; melshatanofy@gmail.com (M.M.E.); yeldez244@yahoo.com (Y.E.K.); mohamedhaggar@gmail.com (M.H.); 3 Department of Biochemistry, Faculty of Science, Alexandria University, Moharam Beik, Alexandria 21547, Egypt; kamkandeel@yaho.com; 4 Department of Medical Laboratory Technology, Faculty of Applied Health Sciences Technology, Pharos University in Alexandria, Alexandria 21311, Egypt; bassma.hassan@pua.edu.eg; 5 Department of Chemistry, Faculty of Science, Taibah University, Al-Madinah Al-Munawarah 30002, Saudi Arabia; aouadmohamedreda@yahoo.fr (M.R.A.); ffs.chem334@gmail.com (F.F.A.); nadjetrezki@yahoo.fr (N.R.); 6 Smart-Health Initiative (SHI) and Red Sea Research Center (RSRC), Division of Biological and Environmental Sciences and Engineering (BESE), King Abdullah University of Science and Technology (KAUST), P.O. Box 4700, Thuwal 23955-6900, Saudi Arabia; mariusz.jaremko@kaust.edu.sa

**Keywords:** tuberculosis, InhA enzyme, 1,2,3- and 1,2,4-Triazoles, in vitro, molecular docking

## Abstract

Tuberculosis (TB) caused by Mycobacterium tuberculosis is still a serious public health concern around the world. More treatment strategies or more specific molecular targets have been sought by researchers. One of the most important targets is M. tuberculosis’ enoyl-acyl carrier protein reductase InhA which is considered a promising, well-studied target for anti-tuberculosis medication development. Our team has made it a goal to find new lead structures that could be useful in the creation of new antitubercular drugs. In this study, a new class of 1,2,3- and 1,2,4-triazole hybrid compounds was prepared. Click synthesis was used to afford 1,2,3-triazoles scaffold linked to 1,2,4-triazole by fixable mercaptomethylene linker. The new prepared compounds have been characterized by different spectroscopic tools. The designed compounds were tested in vitro against the InhA enzyme. At 10 nM, the inhibitors **5b, 5c, 7c, 7d, 7e,** and **7f** successfully and totally (100%) inhibited the InhA enzyme. The IC_50_ values were calculated using different concentrations. With IC_50_ values of 0.074 and 0.13 nM, 7c and 7e were the most promising InhA inhibitors. Furthermore, a molecular docking investigation was carried out to support antitubercular activity as well as to analyze the binding manner of the screened compounds with the target InhA enzyme’s binding site.

## 1. Introduction

**Figure 1:**
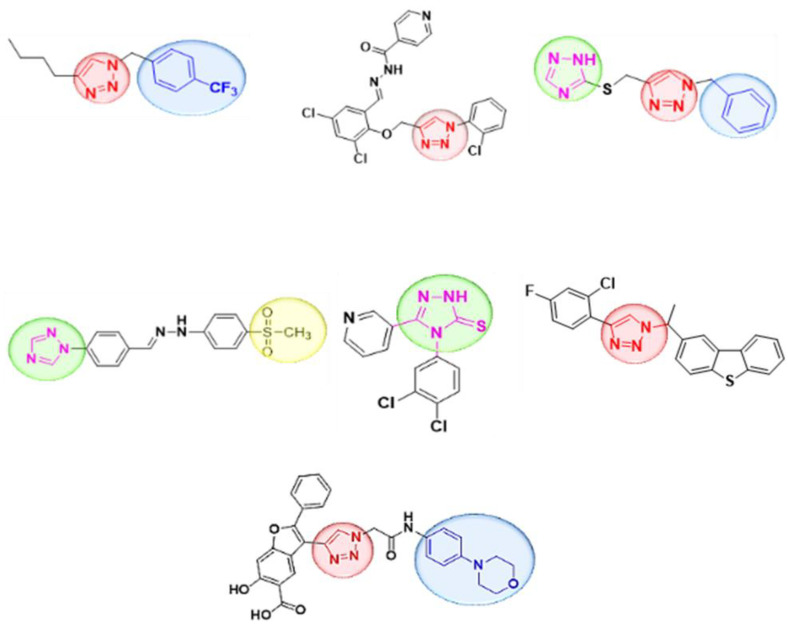
Representative structure of 1,2,3 and/or 1,2,4-triazole derivatives that exhibit anti-tubercular activity.

**Figure 2:**
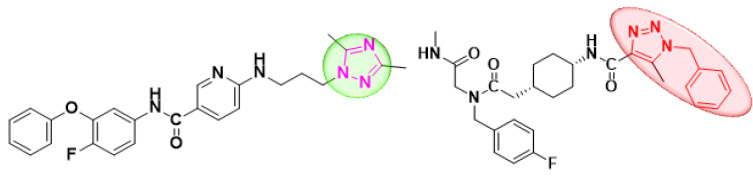
Representative structure of 1,2,3 and/or 1,2,4-triazole derivatives that exhibit InhA inhibitors previously described in the literature.

Nitrogen heterocycles are among the most significant structural components of antimicrobial agents. Among them, 1,4-disubstituted 1,2,3-triazoles, afforded by copper (I) azide alkyne cyclo-addition, portrayed outstanding medicinal chemistry attributes that encouraged the Nobel Prize winner Prof. K. Barry Sharpless to describe them as aggressive pharmacophores [1]. 1,2,3-triazole cores were found in many FDA-approved drugs such as rufinamide (anticonvulsant), TSAO (anti-HIV), cefatrizine (antibiotic), tazobactam (antibacterial), CAI (anticancer), and ribavirin analogs (antiviral) [2,3,4,5,6]. In addition, a literature survey revealed that 1,2,3 and or 1,2,4-triazoles are privileged scaffolds with versatile biological activity, particularly in the area of antimycobacterium drug discovery that utilizes several mechanisms of action, most particularly InhA (Enoyl Acyl Carrier Protein Reductase) inhibitors (Figure 1 and Figure 2) [7,8,9,10,11,12,13,14,15,16,17].

Tuberculosis (TB) is one of the most immense diseases, ranked second after AIDS, which attracted researchers to try to find appropriate treatments. The World Health Organization (WHO) reported Mycobacterium tuberculosis (Mtb) infection as a leading cause of mortality and morbidity worldwide, with 1.6 million deaths annually [18]. Recently, the increasing incidence of multidrug-resistant tuberculosis (MDRTB) and extensively drug-resistant tuberculosis (XDRTB) has worsened the situation [19] and has made TB a more dreadful disease than before. One of the factors that made TB a more complicated infection compared to other microbial infections is their unique cell envelope. The cell envelope contains a protective layer of mycolic acid (which is a saturated chain of β-hydroxy fatty acids along with α-alkyl side chain) [20]. InhA is a NADH-dependent 2-trans enoyl-acyl carrier protein (ACP) reductase of type II fatty acid synthase, which is essential for mycolic acid biosynthesis. There is a portfolio of evidence demonstrating that it is the primary target of the potent and well-known antitubercular drug isoniazid (INH) [18].

**Figure 3:**
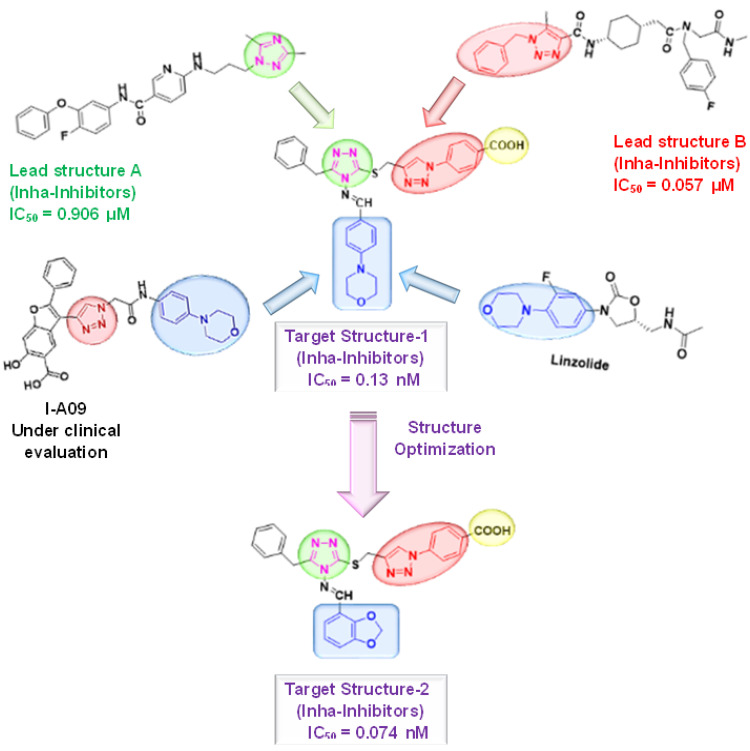
Structure-based rational design of target structures **1, 2**.

A short time ago, there were several drug candidates at different stages of the development pipeline, including one morpholine-containing compound (I-A09) that exerted its antitubercular action through inhibition of protein tyrosine phosphatase B (mPTPB) (Figure 3) [5]. 

**Figure 4:**
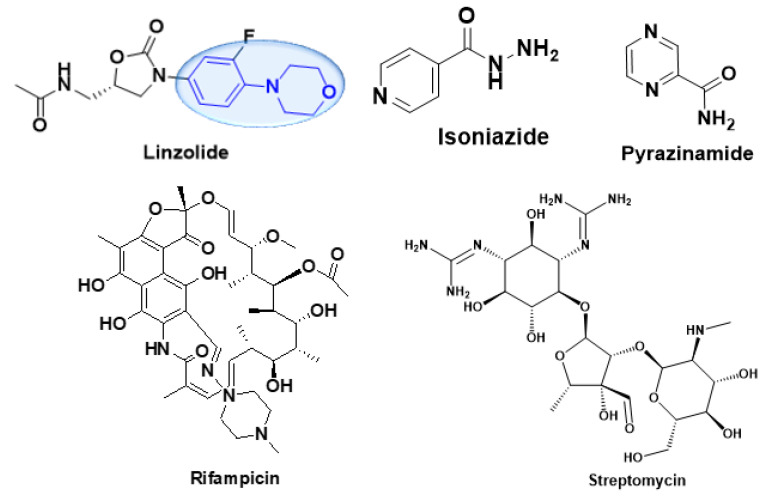
Commercially available anti-tubercular drugs.

Morpholine is a versatile moiety, a privileged pharmacophore, and an extraordinary heterocyclic motif, especially in the field of antimicrobial agents. WHO, for example, recently reclassified a commercially available antimicrobial linezolid as a Group A drug for the treatment of MDRTB and XDRTB [19,20] (Figure 4).

Our lab has committed to discover novel lead structures that might be of value for the development of novel potent antitubercular agents. In the present study, a new set of hybrid derivatives containing 1,2,3-and 1,2,4 triazole moieties were designed based on the structural features of four pleiotropic lead compounds (Figure 3). The newly synthesized compounds were tested in vitro against the mycobacterium tuberculosis InhA enzyme in aspiration of lead structures A and B, which demonstrated remarkable inhibitory activity with IC_50_ values of 0.906 and 0.057 μM, respectively, [4].

The first target structure-1 that exhibit good InhA inhibitory activity with IC_50_ = 0.13 nM was designed by connecting both 1,2,4-triazole scaffold with the click modifiable 1,2,3-triazole via the flexible SCH_2_-bonding and molecular hybridization with morpholine ring achieved by reaction of 4-morpholinobenzaldehyde with 1,2,4-triazole containing compound.

For further optimization and structural versatility, the second target structure was designed, where the 4-phenyl morpholine moiety was replaced by benzo[d][1,3]dioxole, affording a more potent candidate that displayed an IC_50_ of 0.074 nM and was considered the most potent candidate compared to lead structures A, B, and the other tested compounds.

Moreover, the molecular docking study was accomplished both to support the antitubercular activity and to investigate the binding mode of the interactions of the screened compounds with the binding site of the target InhA enzyme.

## 2. Results and Discussion

### 2.1. Chemistry

**Scheme 1:**
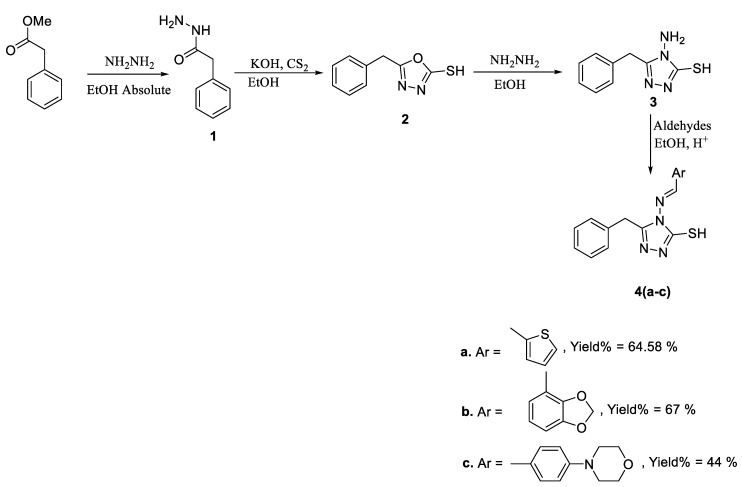
Synthesis of Schiff bases of 4-amino-5-phenylmethyl-1,2,4-triazole-3-thiol **4(a–c)**.

**Scheme 2:**
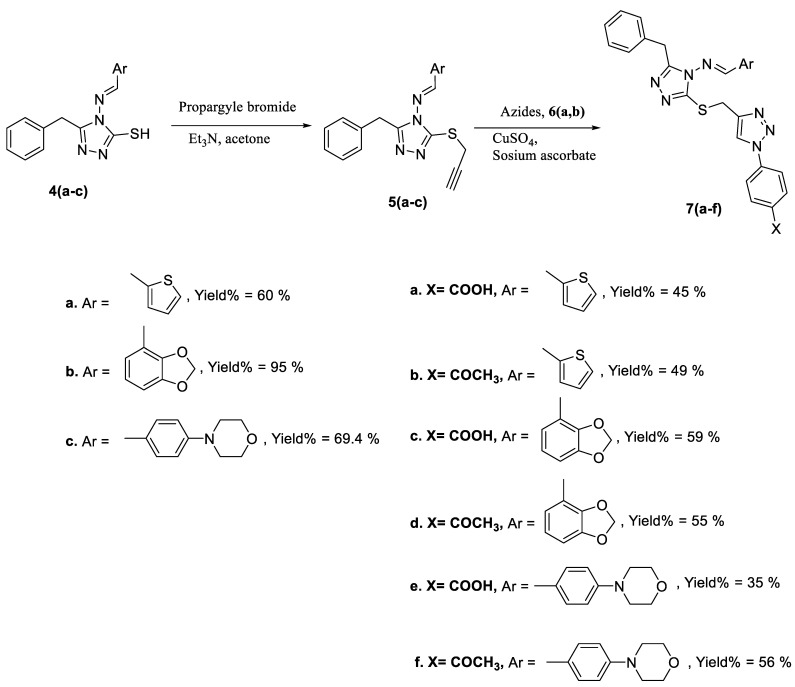
Synthesis of novel 1,2,3-triazoles tethered Schiff bases of 4-amino-5-phenylmethyl-1,2,4-triazole motif **7(a–f)**.

A series of novel 1,2,3-triazoles tethered to a 1,2,4-triazol motif **7(a–f)** were synthesized from 1-amino-1,2,4-triazole **3** as a starting material (Scheme 1). The synthetic strategies adopted in the present work are depicted in Scheme 1 and Scheme 2. The targeted 1,2,4-triazole-1,2,3-triazole molecular conjugates **7(a–f)** were synthesized from three Schiff bases of 4-amino-1,2,4-triazole **4(a–c)**, which in turn were obtained through muli-steps synthesis from the appropriate phenylacetic acid hydrazide **2** according to the reported procedures [21,22] (Scheme 1). The synthesis of Schiff bases of 4-amino-5-phenylmethyl-1,2,4-triazole-3-thiol (**3**) has been achieved successfully by refluxing of the free amine **3** with the appropriate aromatic aldehyde. 

The propargylaion reaction of the free thiol **4(a–c)** with propargyl bromide successfully afforded the corresponding product in quantitative yield. 1,3-Dipolar cycloaddition reaction of propargylated thiol **5(a–c)** with aryl azides **6(a,b)** in the presence of CuSO_4_·5H_2_O and sodium L-ascorbate under stirring at room overnight resulted in the formation of the 4-ayl-1,2,3-triazole derivative **7(a–f)** in yield = 50%.

The success of such 1,3-dipolar cycloaddition was supported by the spectroscopic results of the resulted 1,2,3-triazole **7,** which were in agreement with its proposed structure. The ^1^H-NMR spectrum of **7a** as a representation example displayed two distinguishable singlets at 4.16 and 4.50 ppm of the two nonequivalent methylene groups. The singlet recorded at 8.9 ppm was attributed to the proton of the 1,2,3-triazole residue. Additionally, the ^13^C NMR spectrum revealed no signals on the *sp*-carbon regions, confirming their involvement in the cycloaddition reaction, and one additional signal appeared at 119.91 ppm characteristic for the CH of the triazole. The two methylene carbons (CH_2_) resonated separately in the aliphatic region at 27.48 and 30.71 ppm. 

### 2.2. Biological Evaluation

**Figure 5:**
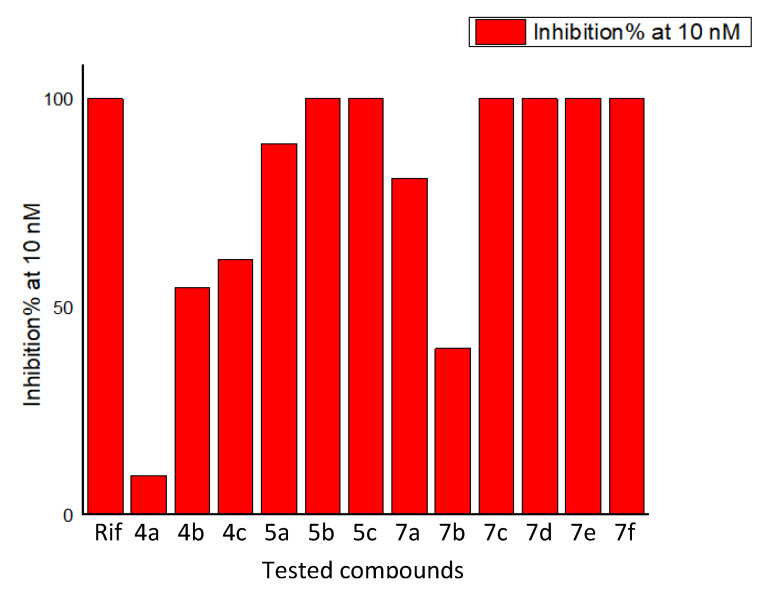
InhA enzyme inhibition percentage at 10 nM of the tested compounds.

A brief screening of the synthesized compounds was tested as a direct InhA enzyme inhibitor, and the InhA inhibition (IC_50_) was calculated. In the present study, **5b**,**c** and **7c–f** successfully and completely (100%) inhibited the InhA enzyme at 10 nM (Figure 5). Different concentrations were prepared and the IC_50_ values were measured. **7c** and **7e** were the most promising agents as InhA inhibitor with IC_50_ = 0.074 and 0.13 nM, respectively. Compared to the known IC_50_ of Rifampicin and Isoniazid, which were reported as 0.8 nM and 54.6 nM, respectively [23], our compounds can pave the way as a new highly active TB drug (Table 1).

### 2.3. Molecular Modeling

#### 2.3.1. Docking Simulations

Compounds **5c, 7d, 7c**, and **7e** were chosen for molecular docking studies into the binding site of inhA based on previous biological evaluation results in order to obtain insight into the hypothesized intermolecular interactions and investigate the possible binding pattern that underpins these drugs’ inhibitory effects. This was accomplished with the help of molecular operating environment software (MOE 2014.0802). The protein data bank provided the X-ray crystal structures of inhA (PDB ID: 4TRO) with its co-crystallized ligand (NAD) [24].

The top-scored conformation with the best binding interactions detected by the MOE search algorithm and scoring function was the basis for the selection of the docking poses. In addition, binding energy scores, formation of binding interaction with the neighboring amino acid residues, and the relative positioning of the docked poses in comparison to the co-crystallized ligands were the factors determining the binding affinities to the binding pockets of the enzymes.

#### 2.3.2. Docking into inhA Active Site

**Figure 6:**
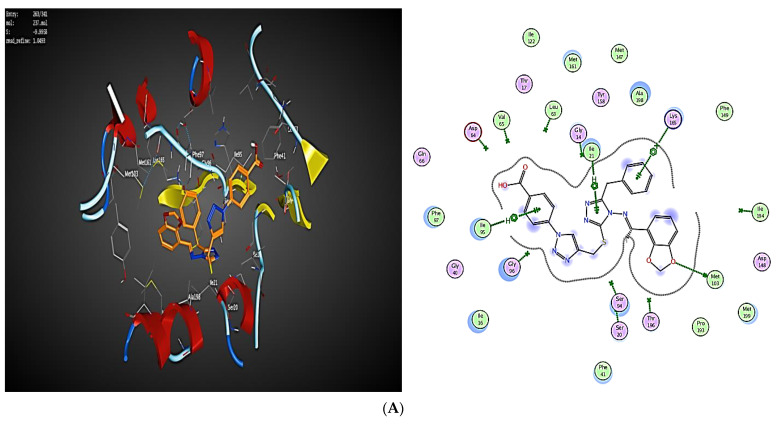

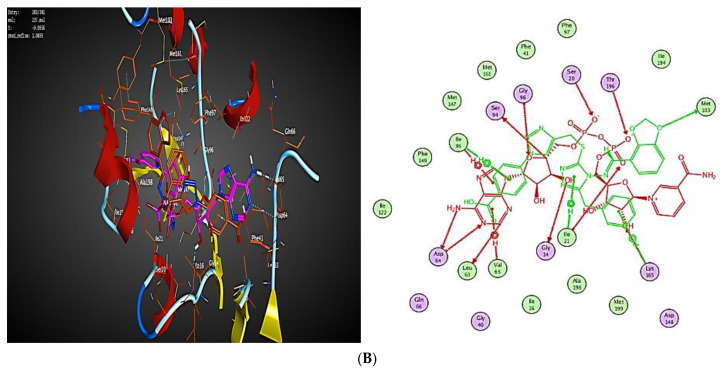
(**A**): Docking and binding pattern of compound **7c** into inhA active site (PDB ID: 4TRO) in 2D (**right panel**) and 3D (**left panel**). (**B**): An overlay of the docked pose of compound **7c** (brown) with the co-crystallized ligand (purple) into inhA active site in 2D (**right panel**) and 3D (**left panel**).

With a binding energy score (S) of −9.99 kcal/mol and a root mean standard deviation (RMSD) of 1.04, compound **7c** was shown to be optimally positioned in the active site of the inhA enzyme in its best-docked pose. It was lodged into the active site through a hydrogen bond of 3.61 Å between oxygen atom of the piperonal moiety and Met103. Furthermore, two hydrophobic interactions with 4.03 and 4.33 Å formed between the aromatic ring part of the 5-benzyl side chain and 1,2,3 triazol-1-yl benzoic acid with Lys165 and Ile95, respectively. In addition, the 1,2,4-triazole ring and Ile21 formed a hydrophobic interaction with 3.87 Å (Figure 6A,B).

**Figure 7:**
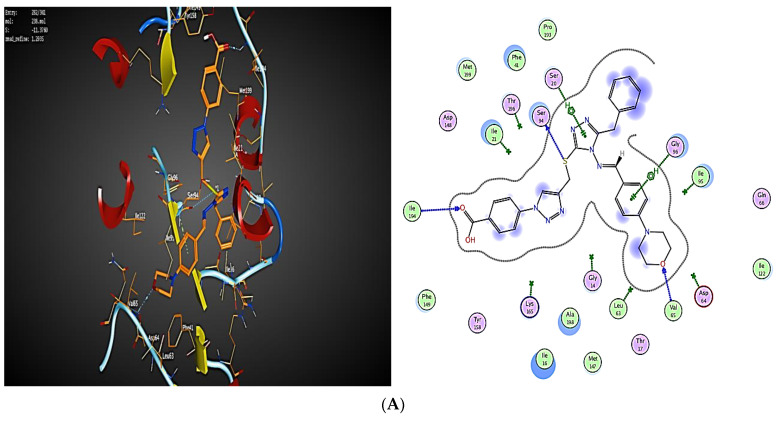

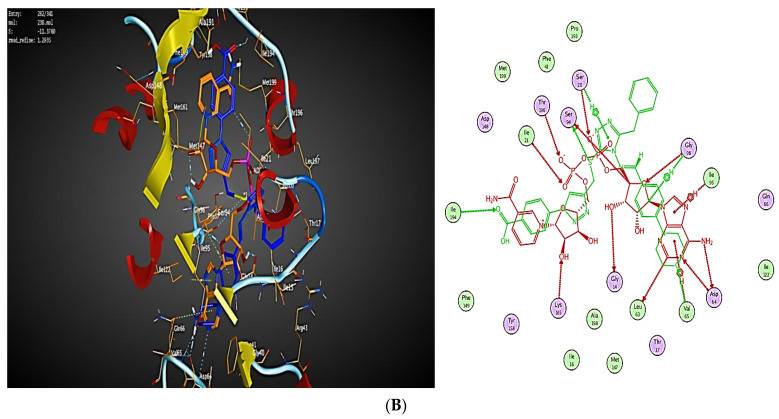
(**A**): Docking and binding pattern of compound **7e** into inhA active site (PDB ID: 4TRO) in 2D (**right panel**) and 3D (**left panel**). (**B**): An overlay of the docked pose of compound **7e** (brown) with the co-crystallized ligand (purple) into inhA active site in 2D (**right panel**) and 3D (**left panel**).

Molecular docking studies of the target compound **7e** displayed (S) −11.7 kcal/mol and (RMSD) of 1.29 revealed that the 4-morpholino ring oxygen forms a hydrogen bond of 3.34 Å with Val65. Furthermore, 1,2,3 triazol-1-yl benzoic acid forms a hydrogen bond of 3.10 Å with Ile194 along the same track, 3-thio atom part in the linker forms a hydrogen bond of 3.70 Å with Ser94. Besides, 2 Hydrophobic π-H bond interactions were encountered between 1,2,4 triazole and 4-benzylidene ring of 3.79 and 4.89 Å with Gly96 and Ser20, respectively (Figure 7A,B).

**Figure 8:**
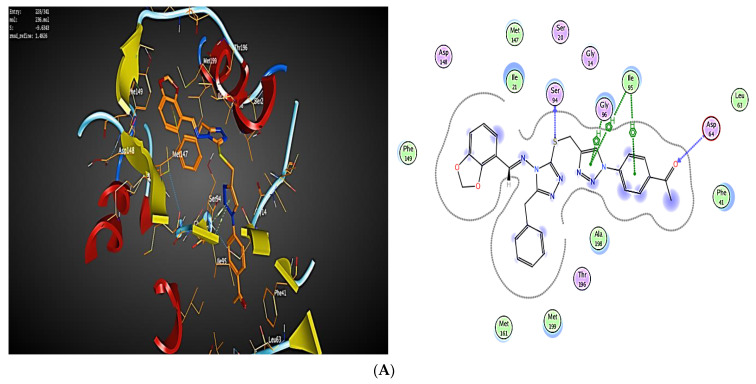

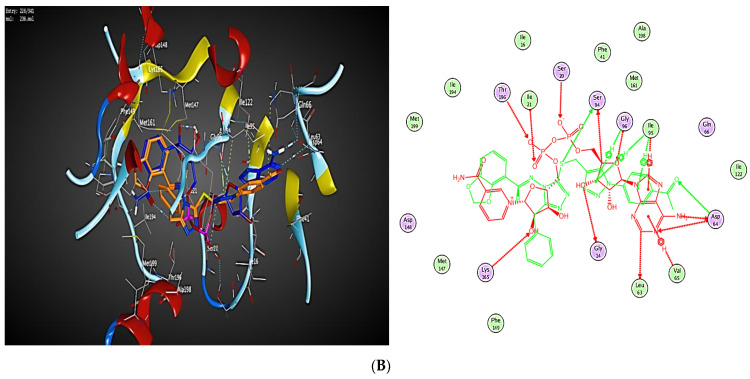
(**A**): Docking and binding pattern of compound **7d** into inhA active site (PDB ID: 4TRO) in 2D (**right panel**) and 3D (**left panel**). (**B**): An overlay of the docked pose of compound **7d** (brown) with the co-crystallized ligand (purple) into inhA active site in 2D (**right panel**) and 3D (**left panel**).

With regard to compound **7d**, which showed (S) −9.63 kcal/mol and (RMSD) of 1.49, H-bonding was observed between the carbonyl oxygen component in 1*H*-1,2,3-triazol-1-yl)phenyl(Ethan-1-one of 3.63 Å and the Asp64. The complex formed was further stabilized by two hydrophobic interactions of 3.96 and 4.62 Å between the previous moiety and Gly96 and Ile95, respectively. This is in addition to the hydrogen bond of 3.24 Å that existed between the 3-thio atom in the methylene thio linker and Ser94 (Figure 8A,B).

**Figure 9:**
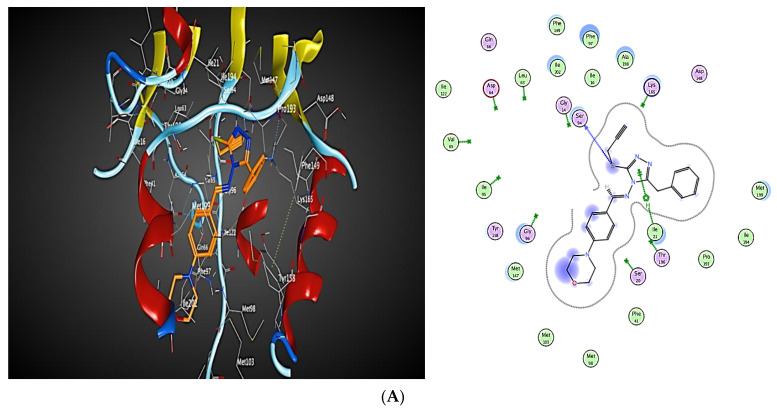

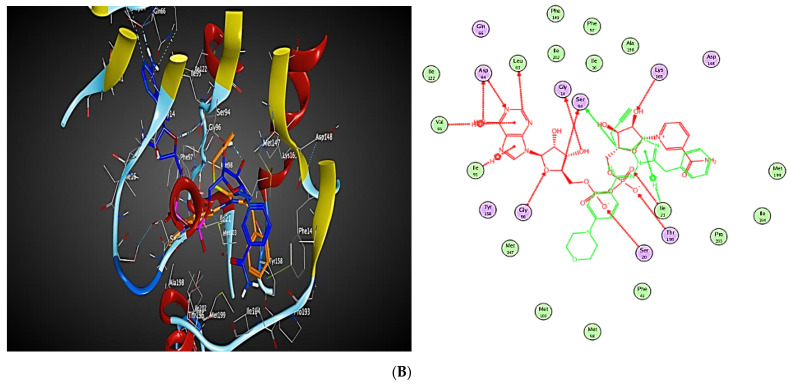
(**A**): Docking and binding pattern of compound **5c** into inhA active site (PDB ID: 4TRO) in 2D (**right panel**) and 3D (**left panel**). (**B**): An overlay of the docked pose of compound **5c** (brown) with the co-crystallized ligand (purple) into inhA active site in 2D (**right panel**) and 3D (**left panel**).

Through a hydrogen bond of 3.70 Å between the thioether atom and Ser94, docking revealed that compound **5c** appropriately occupied the enzyme active site. In addition, the 1,2,4-triazole ring and Ile21 have a hydrophobic interaction of 4.58 Å (Figure 9A,B).

**Figure 10:**
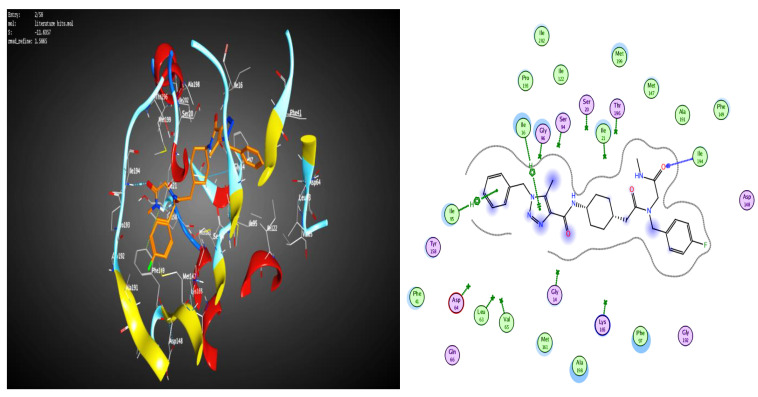
Docking and binding pattern of Lead structure B (brown) into inhA active site in 2D (**right panel**) and 3D (**left panel**).

The reported Inha-Inhibitors Lead structure B IC_50_ = 0.057 μM was selected to docked into 4TRO active site (Figure 10).

Lead structure B was shown (S) −11.63 kcal/mol, and (RMSD) of 1.56 can occupy the active site of the enzyme through hydrogen bond of 3.19 Å between the carbonyl group part in amide side chain and Ile 194. Furthermore, two hydrophobic interaction 4.44, 4.38 Å was observed between 1,2,3-triazole ring and their benzyl moiety with Ile 16 and Ile 95, respectively (Figure 10).

**Figure 11:**
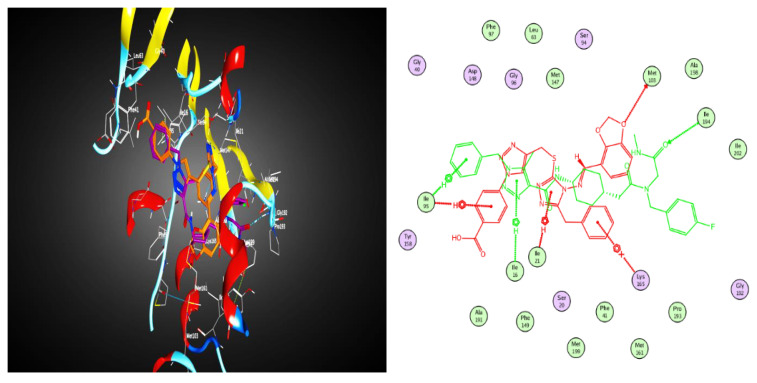
An overlay of the docked pose of compound **7c** (brown) with Lead structure B (purple) into inhA active site in 2D (**right panel**) and 3D (**left panel**).

**Figure 12:**
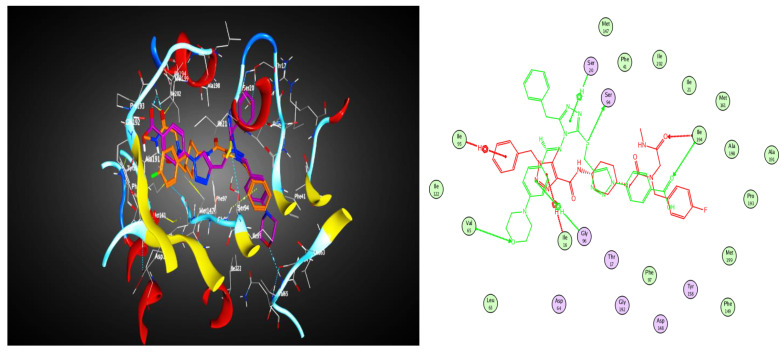
An overlay of the docked pose of compound **7e** (brown) with Lead structure B (purple) into inhA active site in 2D (**right panel**) and 3D (**left panel**).

The examination of the overlay complex between our most potent candidate compounds **7c** and **7e** and lead structure B revealed that Ile 95 in the active site can form the same hydrophobic interaction with compound **7c** and lead structure B. On the other hand, Ile 194 can make the same hydrogen bond with lead structure B and compound **7e** (Figure 11 and Figure 12).

**Figure 13:**
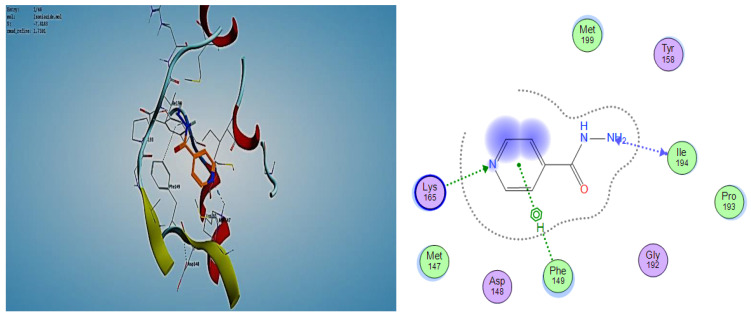
Docking and binding pattern of Isoniazide into inhA active site (PDB ID: 4TRO) in 2D (**right panel**) and 3D (**left panel**).

**Figure 14:**
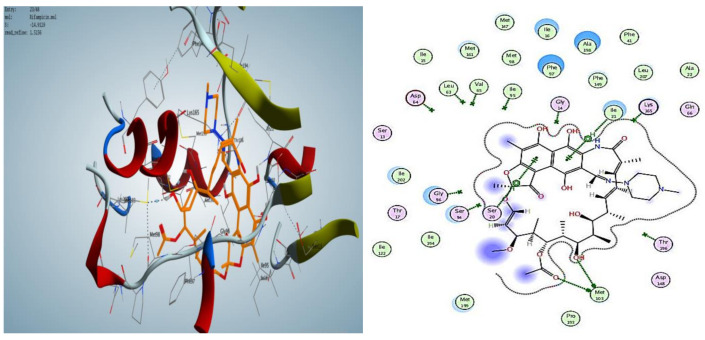
Docking and binding pattern of Rifampicin into inhA active site (PDB ID: 4TRO) in 2D (**right panel**) and 3D (**left panel**).

The Isoniazid molecular docking study with (S) −7.81 kcal/mol and (RMSD) of 1.75 indicate that the pyridine nitrogen and NH_2_ group in the hydrazide moiety form hydrogen bonds of 3.33 and 3.37 Å with Lys165 and Ile194, respectively, while hydrophobic interaction of 4.86 Å showed between pyridine ring and Phe149 (Figure 13). Besides, Rifampicin docking analysis displayed (S) −7.81 kcal/mol and (RMSD) of 1.51 showed two hydrophobic interactions of 4.23 Å and 4.31 Å formed between the naphthyl ring in rifampicin and Ser20 and Ile21, respectively. Furthermore, the carbonyl ester and hydroxyl group of rifampicin side chain form hydrogen bonds of 3.16 and 3.14 Å with Met103 (Figure 13 and Figure 14).

**Figure 15:**
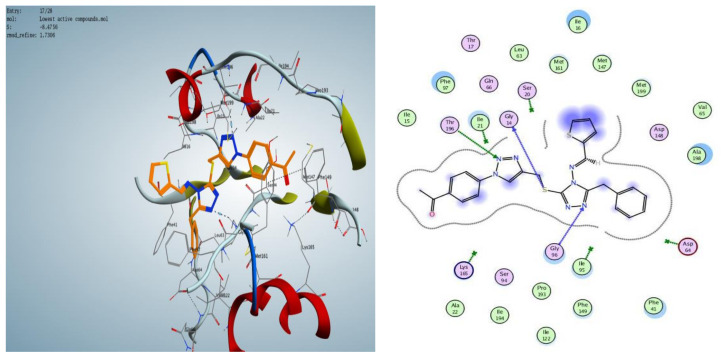
Docking and binding pattern of **7b** into inhA active site (PDB ID: 4TRO) in 2D (**right panel**) and 3D (**left panel**).

Molecular docking studies of the least active **7b** displayed (S) −8.47 kcal/mol and (RMSD) of 1.73 revealed that 1,2,4-triazole nitrogen forms a hydrogen bond of 3.14 Å with Gly96. In addition, 1,2,3-triazole nitrogen forms a hydrogen bond of 2.96 with Thr196 Å while thioether atom forms a hydrogen bond of 2.60 Å with Gly14 (Figure 15).

The high in vitro activity of compounds **7c**, **d** and **e**, as well as the explanation for the lowest activity of compound **7b**, can be explained based on previous docking investigations. Rifampicin’s naphthyl ring forms a hydrophobic interaction with Ile21 and Ser20. Similarly, those two aminoacids form the same type of interaction with the 1,2,4-triazole moiety of compounds **7c**, **d** and **e**. On the other hand, the docking study of the least active **7b** demonstrates that there is no interaction found between previous aminoacids and the **7b** 1,2,4-triazole ring, thus a decline in activity can be predicted. In addition, piperonal oxygen in compound **7c** forms a hydrogen bond with Met103 Rifampicin side chain, sharing the same type of interaction with Met103. Besides, the morpholino phenyl ring in **7e** forms a hydrophobic interaction with Gly96. In contrast, the thiophen ring in **7b** cannot form any type of noncovalent bonding interaction with the surrounding aminoacid inside the active site.

When the activity of the intermediate **5c** is compared to that of **7c**, **d** and **e**, the relevance of the 1,2,3-triazole ring and/or its p-substituted phenyl ring becomes clear. The 1,2,3-triazole ring of Compound **7d** creates a hydrophobic contact with Gly96 and Ile95. In addition, Ile95 forms a hydrophobic interaction with the **7c** and d phenyl rings linked to the 1,2,3-triazole. Furthermore, Ile 194 forms a hydrogen bonding with 1,2,3 triazol-1-yl benzoic acid. Finally, Asp64 can form a hydrogen bond with the carbonyl oxygen component in 1H-1,2,3-triazol-1-yl) phenyl (Ethan-1-one) **7d**.

## 3. Experimental

### 3.1. Chemistry

All reactions were monitored by thin layer chromatography (TLC) on silica gel using 60 F254 aluminum sheets and were visualized under UV lamp at λ = 254 nm. The melting points were recorded and are uncorrected using a Stuart Scientific Melt-Temp apparatus. The IR spectra were recorded on BRUKER spectrometer using KBr disks. The ^1^H NMR (400 MHz) and ^13^C NMR (100 MHz) spectra were recorded using BRUKER spectrometer and TMS as an internal standard to calibrate the chemical shifts (δ) reported in ppm (see Supplementary Materials).

### 3.2. Synthesis of 4-Amino-5-Benzyl-2,4-Dihydro-1,2,4-Triazole-3-Thiol, 3

The reaction of methyl phenylacetate (0.01 mole, 1.50 g) with hydrazine hydrate (0.1 mole, 4.86 mL) in ethanol under reflux for 4 h the benzyl hydrazide **1** was obtained. A mixture of **1** (0.1 mole, 15.01 g) and carbon disulfide (0.15 mole, 9.06 mL) in presence of potassium hydroxide (0.15 mole, 8.41 g) under reflux gave 5-benzyl-1,3,4-oxadiazole-3-thiol **2.** Treatment of **2** (0.01 mole, 1.92 g) with hydrazine hydrate (0.1 mole, 5 g) under reflux gave 4-Amino-5-benzyl-2,4-dihydro-3H-1,2,4-triazole-3-thione. White crystals; Yield = 55%; mp = 172–173 °C, lit. mp = 170 °C [25,26]. 

*4.-(. Arylideneamino)-5-benzyl-2,4-dihydro-3H-1,2,4-triazole-3-thione, **4(a–c).***

#### 3.2.1. General Procedure

The reaction of **3** (0.01 mole, 2.06 g) with aryl aldehyde (0.01 mole) in absolute ethanol with 4 drops conc. H_2_SO_4_ was refluxed for 4 h. A mixture of the reaction was left to cool then poured into cold water. The precipitate was collected via filtration, washed by water, and recrystallized by ethanol.

*5.-benzyl-4-((thiophen-2-ylmethylene)amino)-2,4-dihydro-3H-1,2,4-triazole-3-thione, **4a**.*

Yield = 64.58%, white crystals, mp = 184–186 °C. IR spectrum (KBr, ύ, cm^−1^): 1571 (C=N), 3136 (N-H). ^1^H NMR spectrum (DMSO-d_6_, 400 MHz): δ, ppm = 4.09 (s, 2H, -CH_2_-), 7.22 (t, 2H, J = 4 Hz, Ar-H), 7.30 (d, 4H, J = 4 Hz, Ar-H), 7.74 (d, 1H, J = 4 Hz, Ar-H), 7.91 (d, 1H, J = 4 Hz, Ar-H), 10.21 (s, 1H, -N=CH-), 13.85 (s, 1H, NH). ^13^C NMR spectrum (DMSO, 100 MHz): δ, ppm = 31.19 (Ph-CH_2_), 127.37, 128.94, 129.42, 133.26, 135.48, 135.99, 136.99 (8C, Ar), 150.68 (N=CH) 157.16 (C-triazole), 162.06 (C=S). Anal. Calc. for C_14_H_12_N_4_S_2_ (%): C, 55.98; H, 4.03; N, 18.65; S, 21.35. Found: C, 55.68; H, 4.21, N, 18.90; S, 21.30.

*4.-((benzo[d][dioxol-4-ylmethylene)amino)-5-benzyl-2,4-dihydro-3H-1,2,4-triazole-thione, **4b**.*

Yield = 67%, white crystals, mp = 164 °C. IR spectrum (KBr, ύ, cm^−1^), 1200 (C-O), 1590 (C=N), 3083 (N-H. ^1^H NMR spectrum (CDCl_3_, 400 MHz): δ, ppm = 4.18 (s, 2H, -Ph-CH_2_-), 6.08 (s, 2H, O-CH_2_-O), 7.2–7.4 (m, 8H, Ar-H), 9.84 (s, 1H, -N=CH-), 10.10 (s, 1H, NH). ^13^C NMR spectrum (CDCl_3_, 100 MHz): δ, ppm = 31.43 (Ph-CH_2_), 101.82 (O-CH_2_-O), 106.14, 108.46, 126.67, 126.88, 127.31, 128.72, 128.98, 134.42 (10C, Ar), 148.57 (C-triazole), 151.54 (N=CH), 161.04 (C=S). Anal. Calc. for C_17_H_14_N_4_O_2_S (%): C, 60.34; H, 4.17; N, 16.56. Found: C, 60.04; H, 4.40, N, 16.94.

*(E)-5-benzyl-4-((4-morpholinobenzylidene)amino)-2,4-dihydro-3H-1,2,4-triazole-3-thione, **4c**.*

Yield = 44%, pale brown crystals, mp = 220–222 °C. IR spectrum (KBr, ύ, cm^−1^): 1118 (C-O), 1228 (C-N) 1595 (C=N), 3082 (N-H), ^1^H NMR spectrum (CDCl_3_, 400 MHz): δ, ppm = 3.33 (t, 4H, 2CH_2_, J = 4 Hz, (morpholine)), 3.89 (t, 4H, J = 4 Hz, 2CH_2_(morpholine)), 4.17 (s, 1H, Ph-CH_2_), 6.94 (d, 2H, J = 8 Hz, Ar-H), 7.24–7.31 (m, 5H, Ar-H), 7.74 (d, 2H, J = 8 Hz, Ar-H), 9.91 (s, 1H, -N=CH-), 10.93 (s, 1H, NH). ^13^C NMR spectrum (CDCl_3_, 100 MHz): δ, ppm = 31.46 (Ph-CH_2_), 47.78 (C-N-(morpholine)), 66.56 (C-O-(morpholine)), 114.25, 122.85, 127.23, 128.67, 129.03, 130.47, 131.35, 134.60 (8C, Ar), 151.50 (N=CH), 153.99 (C-triazole), 162.09 (C=S). Anal. Calc. for C_17_H_14_N_4_O_2_S (%): C, 62.31; H, 7.06; N, 18.17. Found: C, 62.47; H, 6.98, N, 18.00.

*1.-Aryl-N-[3-* *benzyl -5-(prop-2-yn-1-yl thio)-4H-1,2,4-triazol-4-yl]methanimine, **5(a–c).***

#### 3.2.2. General Procedure

A mixture of Schiff base derivatives (0.01 mole) and propargyl bromide (0.015 mole, 1.13 mL) in the presence of a catalytic amount of triethylamine (0.015 mole, 0.002 mL) in acetone was refluxed for 8 h. The solvent was removed under reduced pressure and the product was poured into ice. It was crystallized from a mixture of ethanol and water (1:2). 

*(E)-N-(3-benzyl-5-(prop-2-yn-1-ylthio)-4H-1,2,4-triazol-4-yl)-1-(thiophen-2-yl)methanimine, **5a**.*

Yield = 60%, off white crystals, mp = 98 °C. IR spectrum (KBr, ύ, cm^−1^): 1594 (C=N), 3247 (C≡C-H). ^1^H NMR spectrum (DMSO-d_6_, 400 MHz): δ, ppm = 2.49 (t, 1H, J = 2.4 Hz, C≡C-H), 3.94 (d, 2H, J = 2.4 Hz, S-CH_2_), 4.19 (s, 2H, Ph-CH_2_), 7.17–7.27 (m, 6H, Ar-H), 7.75 (d, 1H, J = 4 Hz, Ar-H), 7.98 (d, 1H, J = 4 Hz, Ar-H), 8.96 (s, 1H, -N=CH-). ^13^C NMR spectrum (DMSO, 100 MHz): δ, ppm = 21.39 (S-CH_2_), 30.69 (Ph-CH_2_), 74.87 (C≡C-H), 79.27 (C≡C-H), 126.79, 128.52, 128.74, 128.94, 133.92, 135.61, 135.64, 136.85 (8C, Ar), 144.78 (C-triazole), 151.87 (N=CH), 160.54 (C-triazole). Anal. Calc. for C_17_H_14_N_4_S_2_ (%): C, 59.24; H, 3.73; N, 17.27. Found: C, 59.07; H, 4.00, N, 16.98.

*(E)-1-(benzo[d][1,3]* *dioxol-4-yl)-N-(3-benzyl-5-(prop-2-yn-1-ylthio)-4H-1,2,4-triazol-4-yl)methanimine, **5b**.*

Yield = 95%, buff crystals, mp = 140 °C. IR spectrum (KBr, ύ, cm^−1^), 1260 (C-O), 1623 (C=N), 3219 (C≡C-H). ^1^H NMR spectrum (CDCl_3_, 400 MHz): δ, ppm =2.25 (s, 1H, C≡C-H), 3.98 (d, 2H, -S-CH_2_-), 4.25 (s, 2H, -Ph-CH_2_-), 6.09 (s, 2H, O-CH_2_-O), 6.87 (d, 1H, J = 8 Hz, Ar-H), 7.10 (d, 1H, J = 8 Hz, Ar-H), 7.20–7.27 (m, 6H, Ar-H), 8.24 (s, 1H, -N=CH-). ^13^C NMR spectrum (CDCl_3_, 100 MHz): δ, ppm =22.02 (S-CH_2_), 31.51 (Ph-CH_2_), 72.87 (C≡C-H), 77.95 (C≡C-H), 102.07 (O-CH_2_-O), 106.36, 108.54, 125.91, 127.06, 127.13, 128.68, 128.87, 135.49, 145.18, 148.77 (10C, Ar), 152.23 (C-triazole), 152.75 (N=CH), 164.22 (C-triazole). Anal. Calc. for C_20_H_16_N_4_O_2_S (%): C, 63.81; H, 4.28; N, 14.88. Found: C, 64.07; H, 4.47, N, 15.25.

*(E)-N-(3-benzyl-5-(prop-2-yn-1-ylthio)-1,5-dihydro-4H-1,2,4-triazol-4-yl)-1-(4-morpholinophenyl)methanimine, **5c**.*

Yield = 69.4%, pale brown crystals, mp = 236 °C. IR spectrum (KBr, ύ, cm^−1^): 1169 (C-O), 1363 (C-N) 1589 (C=N), 3227 (C≡C-H). ^1^H NMR spectrum (CDCl_3_, 400 MHz): δ, ppm = 2.24 (s, 1H, C≡C-H), 3.32 (t, 4H, J = 4 Hz, 2CH_2_(morpholine)), 3.85 (t, 4H, J = 4 Hz, 2CH_2_(morpholine)), 3.95 (d, 2H, -S-CH_2_), 4.22 (s, 1H, Ph-CH_2_), 7.1–7.6 (m, 9H, Ar-H), 8.15 (s, 1H, -N=CH-). ^13^C NMR spectrum (CDCl_3_, 100 MHz): δ, ppm = 21.82 (-S-CH_2_), 31.46 (Ph-CH_2_), 46.08 (C-N-(morpholine)), 66.47 (C-O-(morpholine)), 72.77 (C≡C-H), 78.08 (C≡C-H), 114.00, 121.52, 126.96, 128.62, 128.92, 130.87, 135.66, 145.24 (8C, Ar), 152.65 (C-triazole), 154.40 (N=CH), 165.28 (C-triazole). Anal. Calc. for C_23_H_23_N_5_OS (%): C, 64.52; H, 6.65; N, 17.10. Found: C, 64.85; H, 6.33, N, 16.88.

*Aryl azide, **6(a–c).***

A solution of the appropriate aromatic amine (0.015 mole) was dissolved in a mixture of water (10 mL) and sulfuric acid (3 mL). The solution was cooled to 0 °C to which a solution of NaNO_2_ (0.015 mole, 1.06 g) in water (3 mL) was added under constant stirring. Solution of sodium azide (0.0185 mole, 1.20 g) in water (5 mL) was added to the above-mentioned mixture. After additional 15 min of stirring at 0 °C, the formed precipitate was filtered and washed several times with water. Then it was dissolved in ethyl acetate, dried over MgSO_4_, and the solvent was removed under reduced pressure (3). The product was used without crystallization. Yields and melting points of products are listed in Table 2.

*1-(aryl)-N-(3-[[(1-(aryl)-1H-1,2,3-triazol-4-yl)methyl]thio]-5-benzyl-4H-1,2,4-triazol-4-yl)methanimine, **7(a–f).***

#### 3.2.3. General Procedure

A mixture of propargyl derivatives (0.001 mole) and substituted azide (0.003 mole) in 15 mL DMF were stirred for 5 min to form a homogenous solution, then the mixture of (0.2 g) sodium ascorbate and (0.05 g) copper sulfate in 5 mL water was added to the homogenous solution. The reaction mixture was stirred for 24 h. The result was poured into cold water and filtrated off. The product was crystallized from acetonitrile [27].

*(E)-4-(4-(((5-benzyl-4-((thiophen-2-ylmethylene)amino)-4H-1,2,4-triazol-3-yl)thio)methyl)-1H-1,2,3-triazol-1-yl)benzoic acid, **7a**.*

Yield = 45%, off white crystals, mp = 196 °C. IR spectrum (KBr, ύ, cm^−1^): 1493 (C=N), 1700 (C=O), 2481 (COOH). ^1^H NMR spectrum (DMSO-d_6_, 400 MHz): δ, ppm = 4.16 (s, 2H, Ph-CH_2_), 4.50 (s, 2H, S-CH_2_), 7.14–7.24 (m, 6H, Ar-H), 7.71 (d, 1H, J = 4 Hz, Ar-H), 7.79 (d, 1H, J = 4 Hz, Ar-H), 7.98–8.09 (m, 4H, Ar-H), 8.74 (s, 1H, -N=CH-), 8.91 (s, 1H, H-1,2,3triazole), 13.29 (broad, 1H, COOH). ^13^C NMR spectrum (DMSO, 100 MHz): δ, ppm 27.48 (Ph-CH_2_), 30.71 (S-CH_2_), 119.91 (C-triazole), 122.02, 126.72, 128.46, 128.64, 128.70, 131.29, 131.29, 133.80 (12C, Ar), 135.60, 136.72, 139.36 (C-triazole), 160.29 (COOH). Anal. Calc. for C_24_H_19_N_7_O_2_S_2_ (%): C, 57.47; H, 3.82; N, 19.55. Found: C, 57.16; H, 3.98; N, 19.85.

*(E)-1-(4-(4-(((5-benzyl-4-((thiophen-2-ylmethylene)amino)-4H-1,2,4-triazol-3-yl)thio)methyl)-1H-1,2,3-triazol-1-yl)phenyl(Ethan-1-one, **7b**.*

Yield = 49%, off white crystals, mp = 196 °C. IR spectrum (KBr, ύ, cm^−1^): 157c (C=N), 1677 (C=O). ^1^H NMR spectrum (CDCl_3_, 400 MHz): δ, ppm = 2.65 (s, 3H, COCH_3_), 4.18 (s, 2H, Ph-CH_2_), 4.62 (s, 2H, S-CH_2_), 7.13–7.28 (m, 6H, Ar-H), 7.41 (d, 1H, J = 4.4 Hz, Ar-H), 7.61 (d, 1H, J = 4.4 Hz, Ar-H), 7.81 (d, 2H, J = 6.4 Hz, Ar-H), 8.08 (d, 2H, J = 6.4 Hz, Ar-H), 8.27 (s, 1H, -N=CH-), 8.42 (s, 1H, H-1,2,3triazole). ^13^C NMR spectrum (CDCl_3_, 100 MHz): δ, ppm= 26.59 (CH_3_), 27.18 (Ph-CH_2_), 31.47 (S-CH_2_), 119.97 (C-triazole), 121.56, 126.93, 128.12, 128.55, 128.86, 129.90, 132.68 (12C, Ar), 135.09, 136.70, 139.80 (C-triazole), 156.85 (N=CH), 196.49 (C=O). Anal. Calc. for C_25_H_21_N_7_OS_2_ (%): C, 60.10; H, 4.24; N, 19.63. Found: C, 60.47; H, 4.40; N, 19.81.

*(E)-4-(4-(((4-((benzo[d][1,3]* *dioxol-4-ylmethylene)amino)-5-benzyl-4H-1,2,4-triazol-3-yl)thio)methyl)-1H-1,2,3-triazol-1-yl)benzoic acid, **7c.***

Yield = 59%, off white powder crystals, mp = 188 °C. IR spectrum (KBr, ύ, cm^−1^), 1266 (C-O), 1448 (C=N), 1695 (C=O), 2465 (COOH). ^1^H NMR spectrum (DMSO-d_6_, 400 MHz): δ, ppm =4.19 (s, 2H, -Ph-CH_2_-), 4.49 (s, 2H, -S-CH_2_-), 6.13 (s, 2H, O-CH_2_-O), 7.02 (d, 1H, J = 8 Hz, Ar-H), 7.18–8.11 (m, 11H, Ar-H), 8.58 (s, 1H, H-1,2,3-triazole) 8.71 (s, 1H, -N=CH-), 13.11 (broad, 1H, COOH). ^13^C NMR spectrum (DMSO, 100 MHz): δ, ppm =28.22 (Ph-CH_2_), 31.15 (S-CH_2_), 102.59 (O-CH_2_-O), 106.29, 109.10, 122.38, 126.29, 127.12, 127.41, 128.89, 129.07, 136.25 (14C, Ar), 120.59, 139.78, 144.70, 148.56 (C-triazole), 152.08 (N=CH), 166.04 (COOH). Anal. Calc. for C_27_H_21_N_7_O_4_S (%): C, 60.10; H, 3.92; N, 18.17. Found: C, 60.7c; H, 4.15, N, 18.41.

*(E)-1-(4-(4-(((4-((benzo[d][1,3]* *dioxol-4-ylmethylene)amino)-5-benzyl-4H-1,2,4-triazol-3-yl)thio)methyl)-1H-1,2,3-triazol-1-yl)phenyl(Ethan-1-one, **7d.***

Yield = 55%, off white crystals, mp = 196 °C. IR spectrum (KBr, ύ, cm^−1^): 1210 (C-O), 1450 (C=N), 1672 (C=O). ^1^H NMR spectrum (DMSO-d_6_, 400 MHz): δ, ppm =2.49 (s, 3H, COCH_3_), 4.21 (s, 2H, -Ph-CH_2_-), 4.49 (s, 2H, -S-CH_2_-), 6.11 (s, 2H, O-CH_2_-O), 7.02 (d, 1H, J = 6.4 Hz, Ar-H), 7.14–8.10 (m, 11H, Ar-H), 8.12 (s, 1H, H-1,2,3-triazole) 8.57 (s, 1H, -N=CH-). ^13^C NMR spectrum (DMSO, 100 MHz): δ, ppm = 26.86 (CH_3_), 27.73 (Ph-CH_2_), 30.62 (S-CH_2_), 102.19 (O-CH_2_-O), 105.68, 108.68, 125.70, 126.74, 127.19, 128.47, 128.60, 130.04 (14C, Ar), 119.62, 139.78, 144.70, 148.65 (C-triazole). 165.95 (N=CH), 196.96 (C=O). Anal. Calc. for C_28_H_23_N_7_O_3_S (%): C, 62.56; H, 4.31; N, 18.24. Found: C, 62.49; H, 4.46, N, 18.16.

*(E)-4-(4-(((5-benzyl-4-((4-morpholinobenzylidene)amino)-4H-1,2,4-triazol-3-yl)thio)methyl)-1H-1,2,3-triazol-1-yl)benzoic acid, **7e**.*

Yield = 35%, pale yellow powder, mp = 192 °C. IR spectrum (KBr, ύ, cm^−1^): 1232 (C-O), 1588 (C=N), 1706 (C=O), 2615 (COOH). ^1^H NMR spectrum (DMSO-d_6_, 400 MHz): δ, ppm = 3.29 (s, 4H, 2CH_2_(morpholine)), 3.74 (s, 4H, 2CH_2_(morpholine), 4.19 (s, 1H, Ph-CH_2_), 4.22 (s, 2H, -S-CH_2_), 6.98–8.33 (m, 13H, Ar-H), 8.45 (s, 1H, H-1,2,3-triazole), 8.71 (s, 1H, -N=CH-), 13.00 (broad, 1H, COOH). ^13^C NMR spectrum (DMSO, 100 MHz): δ, ppm = 28.08 (Ph-CH_2_), 31.12 (-S-CH_2_), 47.22 (C-N-(morpholine)), 66.27 (C-O-(morpholine)), 114.13, 122.51, 127.14, 128.89, 129.02, 131.05 (12C, Ar), 121.15, 136.12, 139.48, 145.09 (C-triazole), 154.57 (N=CH), 167.40 (COOH). Anal. Calc. for C_30_H_28_N_8_O_3_S (%): C, 62.05; H, 4.86; N, 19.30. Found: C, 61.75; H, 4.60, N, 19.24.

*(E)-1-(4-(4-(((5-benzyl-4-((4-morpholinobenzylidene)amino)-4H-1,2,4-triazol-3-yl)thio)methyl)-1H-1,2,3-triazol-1-yl)phenyl(Ethan-1-one, **7f**.*

Yield = 56%, pale brown crystals, mp = 198–200 °C. IR spectrum (KBr, ύ, cm^−1^): 1554 (C=N), 1676 (C=O). ^1^H NMR spectrum (DMSO-d_6_, 400 MHz): δ, ppm = 2.64 (s, 3H, CH_3_), 3.29 (s, 4H, 2CH_2_(morpholine)), 3.73 (s, 4H, 2CH_2_(morpholine), 4.17 (s, 1H, Ph-CH_2_), 4.50 (s, 2H, -S-CH_2_), 6.98 (d, 2H, J = 8 Hz, Ar-H), 7.18–7.24 (m, 5H, Ar-H), 7.61 (d, 2H, J = 8 Hz, Ar-H), 7.98 (d, 2H, J = 8 Hz, Ar-H), 8.13 (d, 2H, J = 8 Hz, Ar-H), 8.46 (s, 1H, H-1,2,3-triazole), 8.75 (s, 1H, -N=CH-). ^13^C NMR spectrum (DMSO, 100 MHz): δ, ppm = 27.26 (CH_3_), 27.96 (Ph-CH_2_), 31.15 (-S-CH_2_), 47.25 (C-N-(morpholine)), 66.27 (C-O-(morpholine)), 114.16, 121.31, 122.43, 127.11, 128.88, 129.07, 130.49, 130.99, 136.36, 136.88, 139.9 (12C, Ar), 120.16, 139.91, 144.82, 154.54 (C-triazole), 166.92 (N=CH), 197.34 (C=O). Anal. Calc. for C_31_H_30_N_8_O_2_S (%): C, 64.34; H, H,5.23; N, 19.36. Found: C, 64.34; H, 5.44, N, 19.7c.

#### 3.2.4. Enzymatic Inhibition Experiments

M. tuberculosis InhA was overexpressed in E. coli while isoniazid and NADH were obtained from Sigma–Aldrich. The concentration of the pool INH–NAD was determined on the basis of ε_330_ equaling 6900 M^−1^ cm^−1^. The substrate 2-trans-decenoyl-CoA concentration was determined on the basis of ε_260_ equaling 22 600 M^−1^ cm^−1^.

For the inhibition assays with InhA, the pre-incubation reactions were performed in 80 μL (total volume) of 30 mM PIPES buffer solution, 150 mM NaCl, pH 6.8 at 25 °C containing 70 nM InhA and the tested compounds (at different concentrations). DMSO was used as a co-solvent and its final concentration was 0.5%. After 2 h of pre-incubation, the addition of 35 μM substrate (trans-2-decenoyl-CoA) and 200 μM cofactor (NADH) initiated the reaction which was measured at 25 °C and at 340 nm (oxidation of NADH) using a spectrophotometer (PG-T80, UK). Control reactions were done under the same conditions, but without the ligands. The pool of INH–NAD adducts was used as a positive control. The initial rates of the reactions were calculated. Rifampicin was used as a reference commercially known drug. The inhibition percentage of each compound was measured at 10 nM and the compounds’ inhibitory activity was expressed as the IC_50_ inhibition of InhA activity with respect to the control experiments [28].

##### Docking Study

Computer-aided docking experiments were performed using Molecular Operating Environment (MOE 2014.0802) software (Chemical Computing Group, Montreal, QC, Canada).

##### Preparation of the Protein Crystal Structures

The protein data bank provided the X-ray crystal structures of inhA (PDB ID: 4TRO) with its co-crystallized ligand (NAD).

Redundant chains, water molecules, and any surfactants were discarded, explicit hydrogen atoms were added to the receptor complex structure, and partial charges were calculated. The preparation was completed with structure preparation module employing protonated 3D function. The co-crystal ligands were extracted from their corresponding proteins and used as reference molecules for the validation study. 

##### Preparation of the Selected Compounds for Docking 

The target compounds were constructed using the builder module of MOE. The compounds were then collected in a database and prepared by adding hydrogens, calculating partial charges and energy minimizing using Force field MMFF94x.

The top-scored conformation with the best binding interactions detected by the MOE search algorithm and scoring function was the basis for the selection of the docking poses. In addition, binding energy scores, formation of binding interaction with the neighboring amino acid residues, and the relative positioning of the docked poses in comparison to the co-crystallized ligands were the factors determining the binding affinities to the binding pockets of the enzyme.

## 4. Conclusions

A new set of hybrid derivatives containing 1,2,4-and click modifiable 1,2,3 triazole moieties were designed and synthesized in order to target M. tuberculosis’ enoyl-acyl carrier protein reductase InhA. In vitro study results revealed a successful and complete (100%) inhibition for some compounds at certain concentration. Of the investigated compounds, **5b**, **5c**, **7c**, **7d**, **7e**, and **7f** completely inhibited the InhA enzyme at 10 nM. Different concentrations were used to calculate the IC_50_ values. The results showed that compounds **7c** and **7e** were the most promising InhA inhibitors, with IC_50_ values of 0.074 and 0.13 nM, respectively, so our compounds have the potential to pave the way for new highly active anti-TB medications.

## Figures and Tables

**Table 1:** The inhibitory effect (IC_50_) of the tested compounds against InhA.

Tested Compounds	IC_50_ (nM)
**4a**	53.2
**4b**	9.16
**4c**	8.16
**5a**	5.61
**5b**	0.66
**5c**	0.23
**7a**	6.18
**7b**	12.5
**7c**	0.074
**7d**	0.18
**7e**	0.13
**7f**	0.25

**Table 2:** M.Ps and yield % of Substituted phenyl azide, **6(a–c)**.

ID.	R^1^	M.P. (°C)	M.P. (°C) [Lit]	Yield (%)
**6a**	COOH	190–193	(190) [27]	80
**6b**	COCH_3_	176–178	(178–180) [27]	77

## Data Availability

The data that support the findings of this study are available from the corresponding author upon reasonable request.

## Supplementary Materials

The following are available online at https://www.mdpi.com/article/10.3390/ijms23094706/s1.
