# Supplementary material for: Novel Hybrid 1,2,4- and 1,2,3-Triazoles Targeting Mycobacterium Tuberculosis Enoyl Acyl Carrier Protein Reductase (InhA): Design, Synthesis, and Molecular Docking
Source: Int J Mol Sci. 2022 Apr 24;23(9):4706. doi: 10.3390/ijms23094706 (PMC9103244; doi:10.3390/ijms23094706)
Supplement: Supplementary file 1 [file ijms-23-04706-s001.zip › ijms-1665396-supplementary.pdf]

SUPPORTING INFORMATION

Novel Hybrid 1,2,4- and 1,2,3-Triazoles Targeting Mycobacterium Tuberculosis Enoyl Acyl Carrier Protein Reductase (InhA): Design, Synthesis and Molecular Docking

Maged A. El Sawy [1*], Maram M. Elshatanofy [2], Yeldez El Kilany [2], Kamal Kandeel [3], Bassma H Elwakil[4], Mohamed Hagar [2] Mohamed Reda Aouad[5], Fawzia Faleh Albelwi [5], Nadjet Rezki [5], Mariusz Jaremko [6] and El Sayed H. El Ashry [2*]

1. Department of Pharmaceutical Chemistry, Faculty of Pharmacy. Pharos University, 21311, Alexandria, Egypt
2. Department of Chemistry, Faculty of Science, Alexandria University, Alexandria 21321, Egypt.
3. Department of Biochemistry, Faculty of Science, Alexandria University, Moharam Beik, 21547, Alexandria, Egypt, kamkandeel@yaho.com.
4. Department of Medical laboratory technology, Faculty of Applied Health Sciences Technology, Pharos University in Alexandria, Alexandria, Egypt. bassma.hassan@pua.edu.eg
5. Department of Chemistry, Faculty of Science, Taibah University, Al-Madinah Al-Munawarah 30002, Saudi Arabia
6. Biological and Environmental Sciences & Engineering Division (BESE), King Abdullah University of Science and Technology (KAUST), Thuwal 23955-6900, Saudi Arabia; mariusz.jaremko@kaust.edu.sa

* Correspondence: MAE, maged.elsawy@pua.edu.eg; EEA, eelashry60@hotmail.com

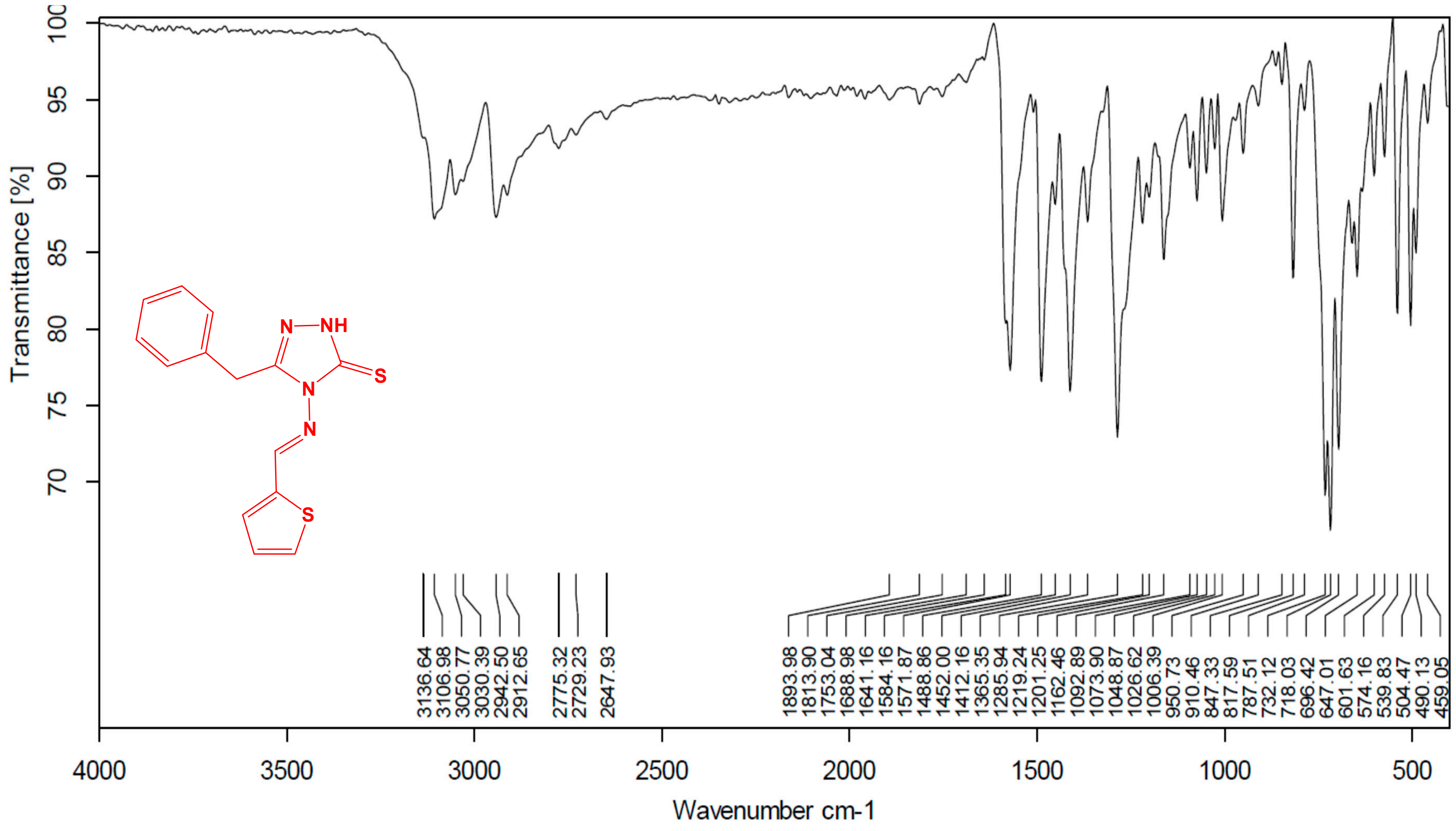

Figure S1: IR Spectrum of compound 4a

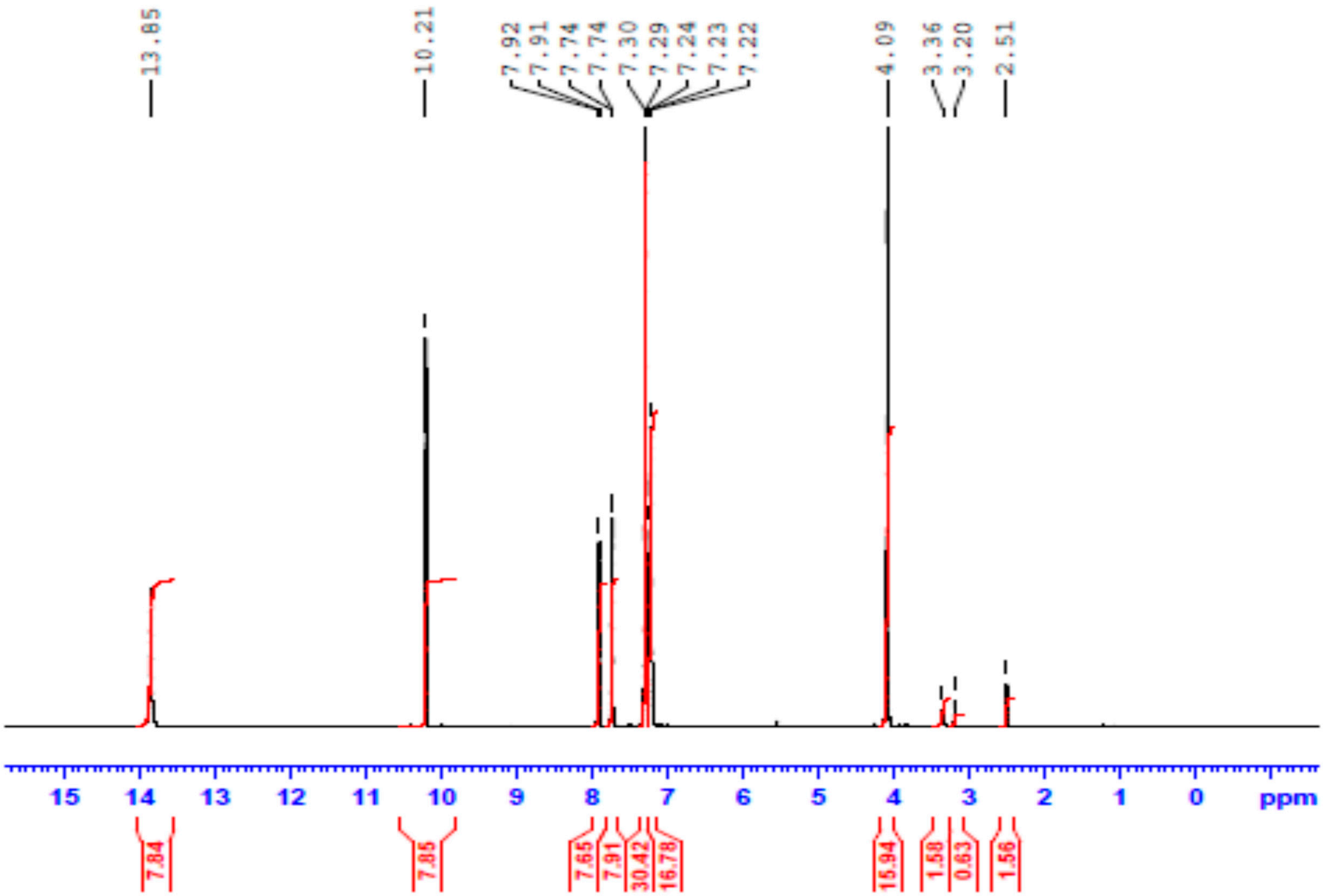

Figure S2: ^{1}H NMR Spectrum of compound 4a

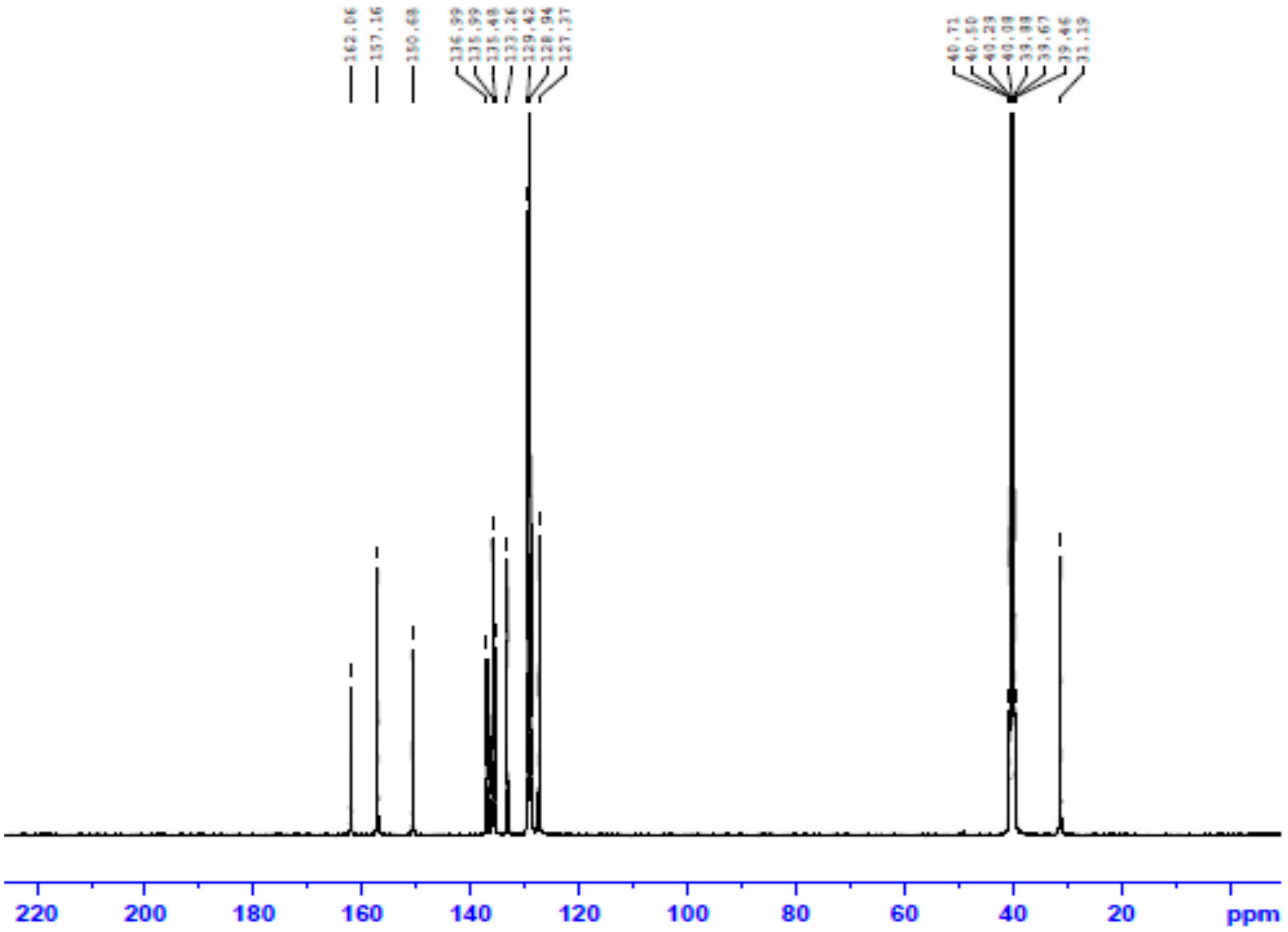

Figure S3: ^{13}**C NMR Spectrum of compound 4a**

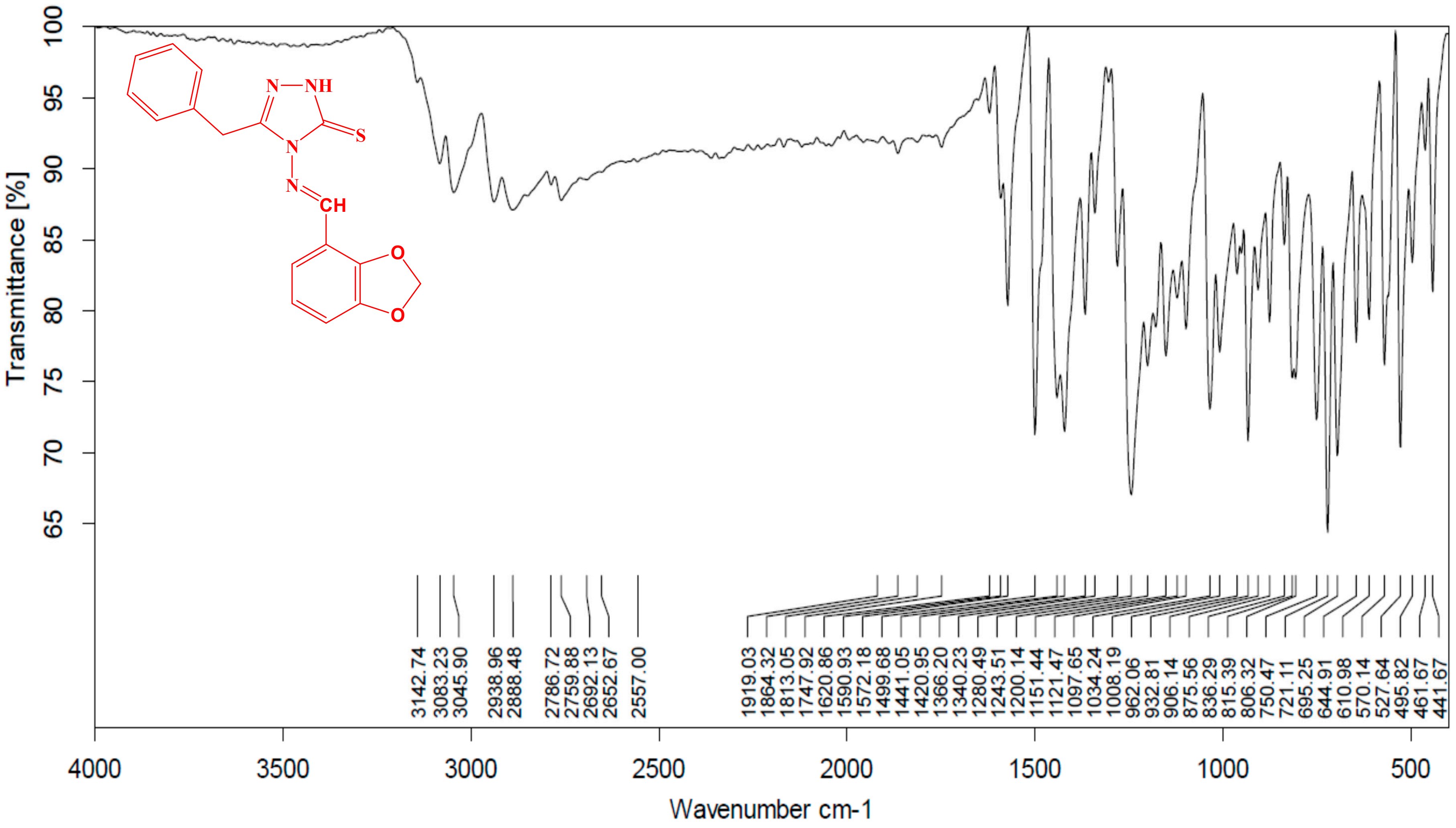

Figure S4: IR Spectrum of compound 4b

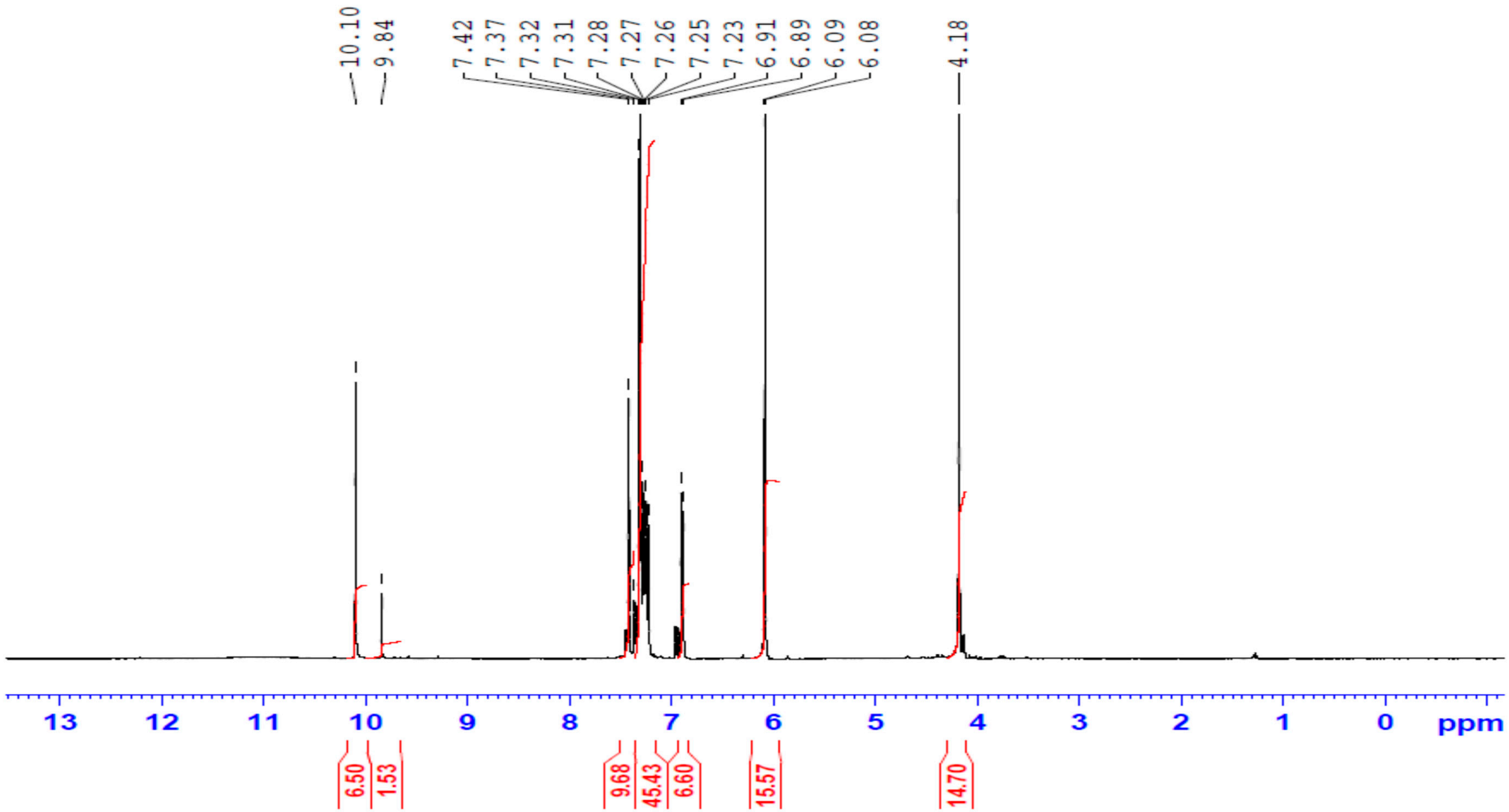

Figure S5: ^{1}H NMR Spectrum of compound 4b

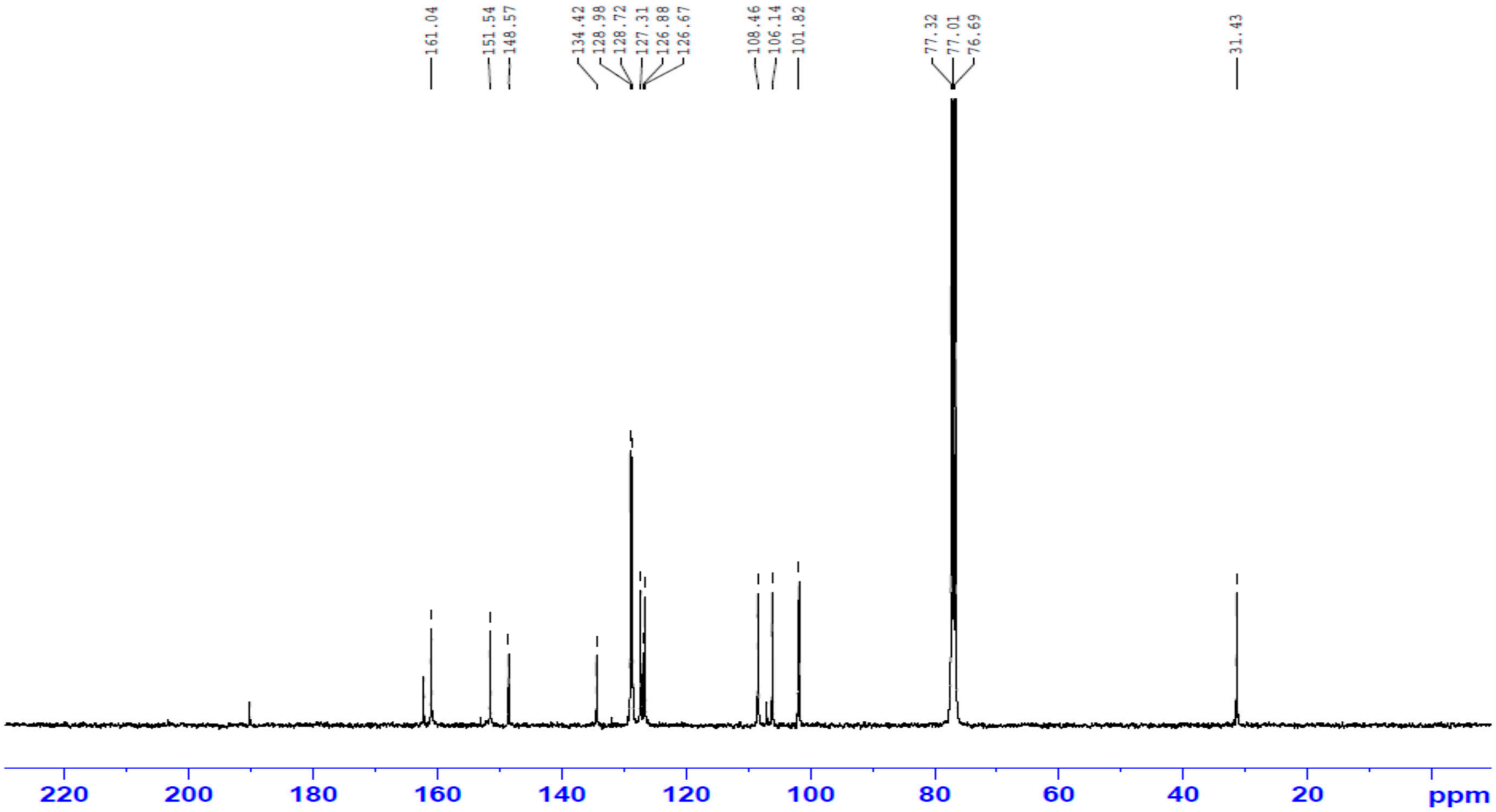

Figure S6: ^{13}C **NMR Spectrum of compound 4b**

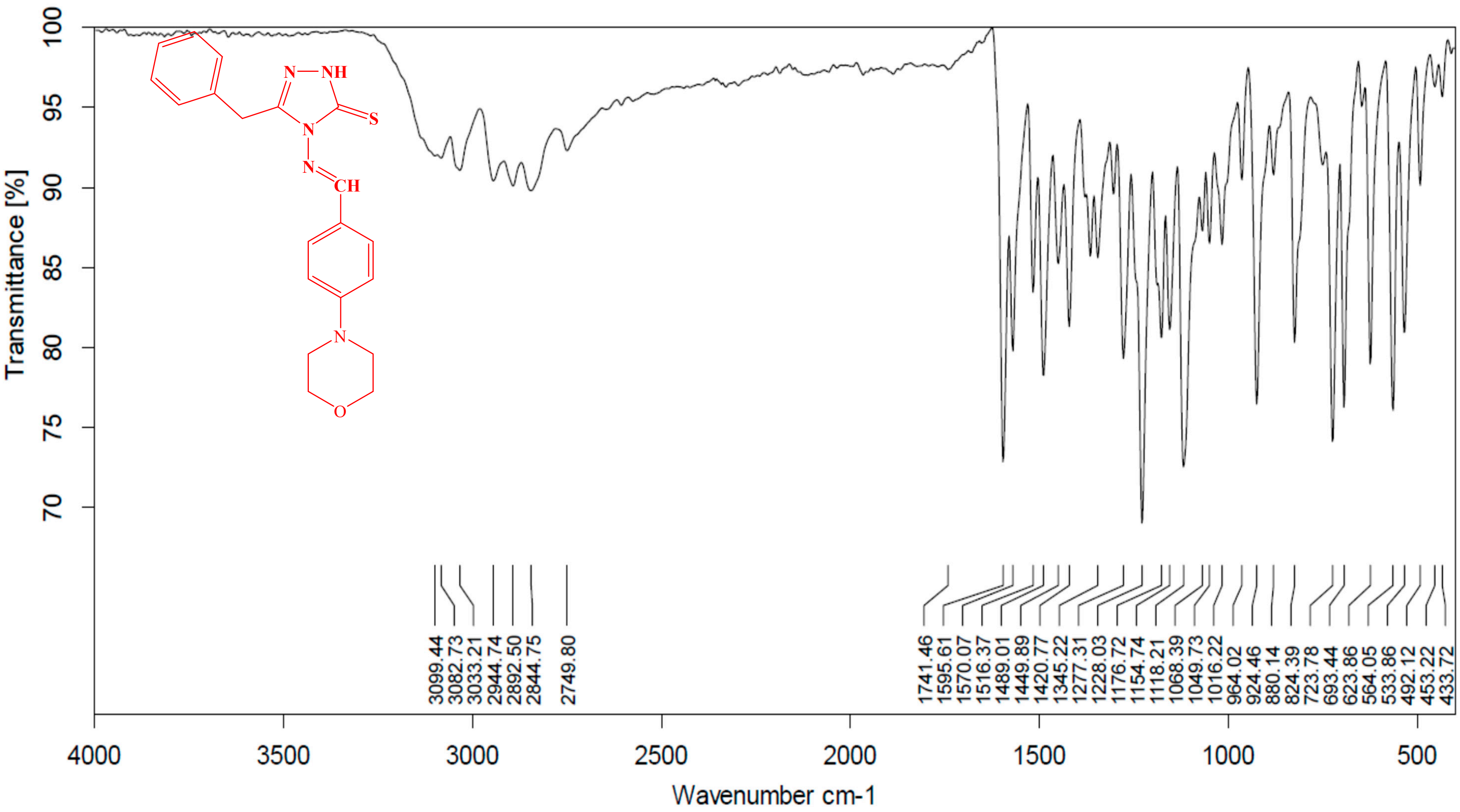

Figure S7: IR Spectrum of compound 4c

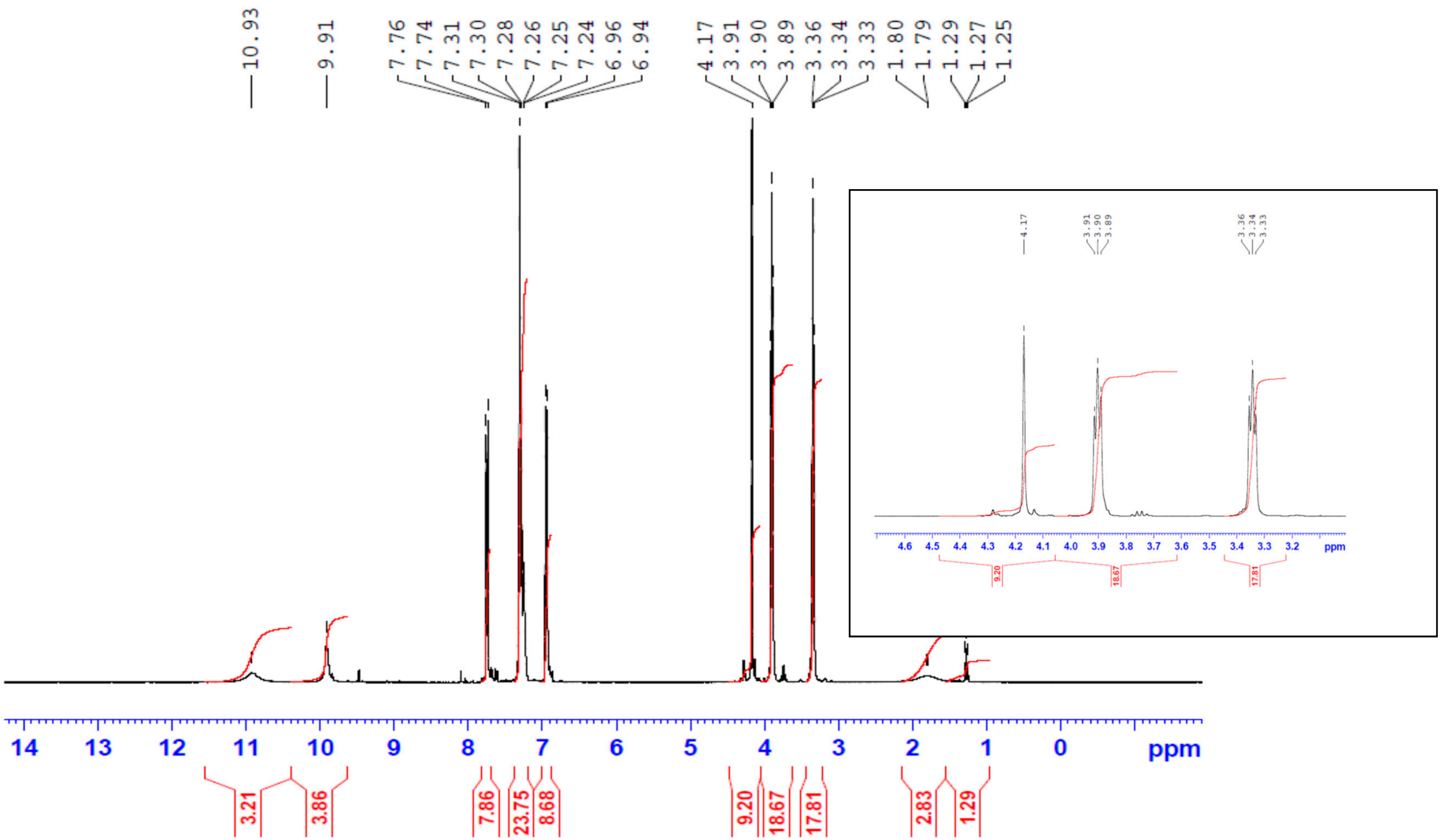

Figure S8: ^{1}H NMR Spectrum of compound 4c

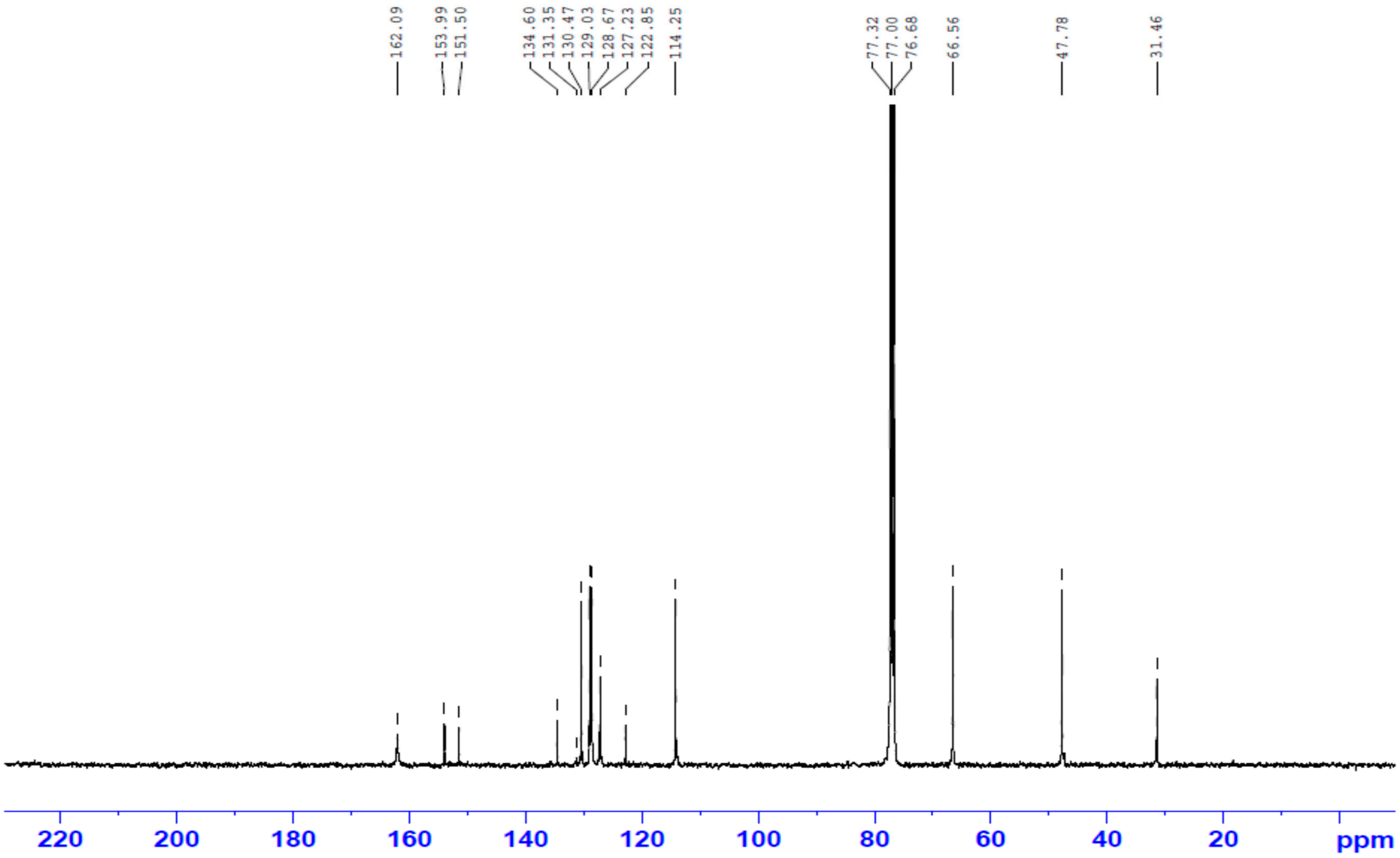

Figure S9: ^{13}C NMR Spectrum of compound 4c

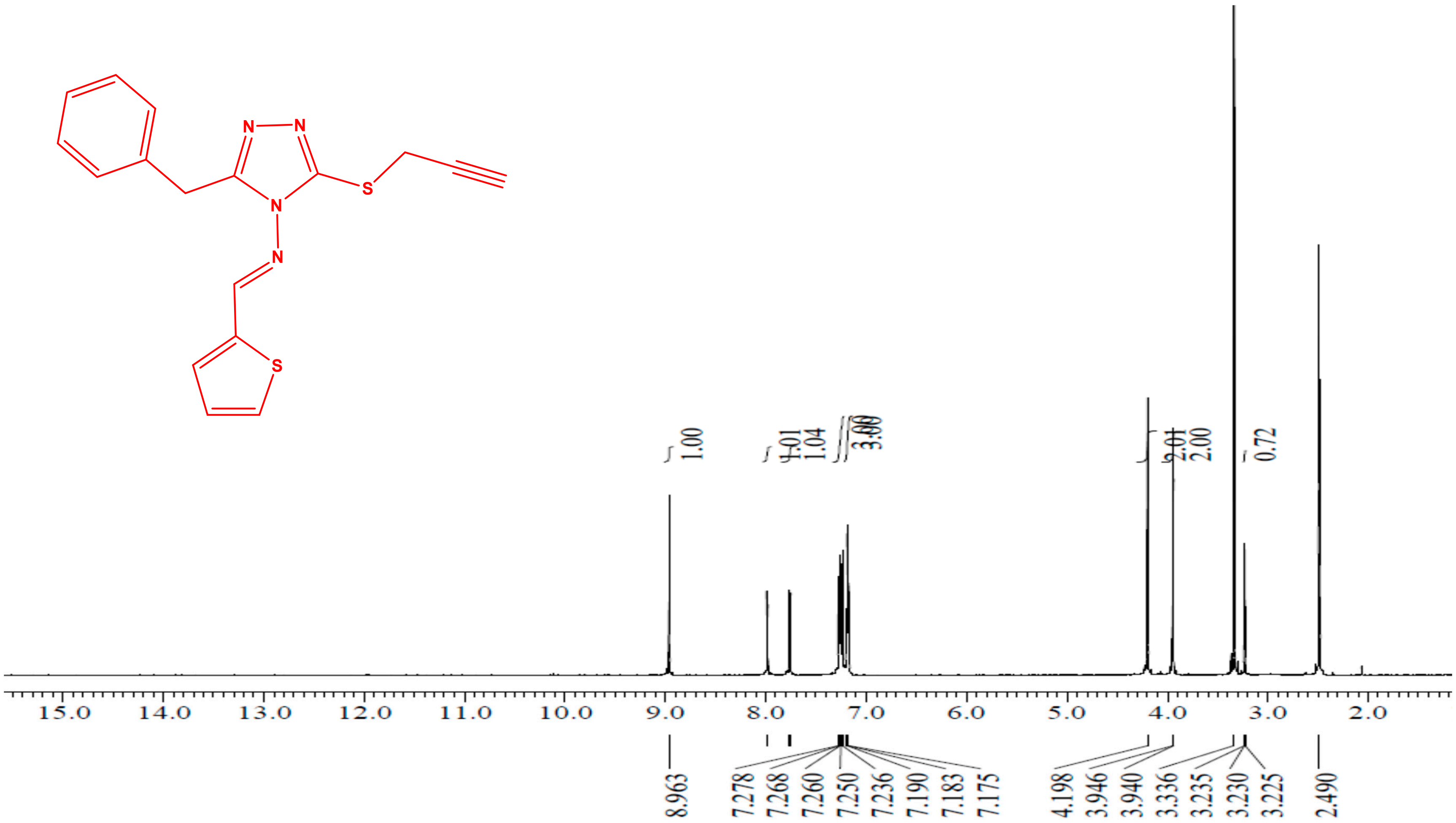

Figure S10: ^{1}H NMR Spectrum of compound 5a

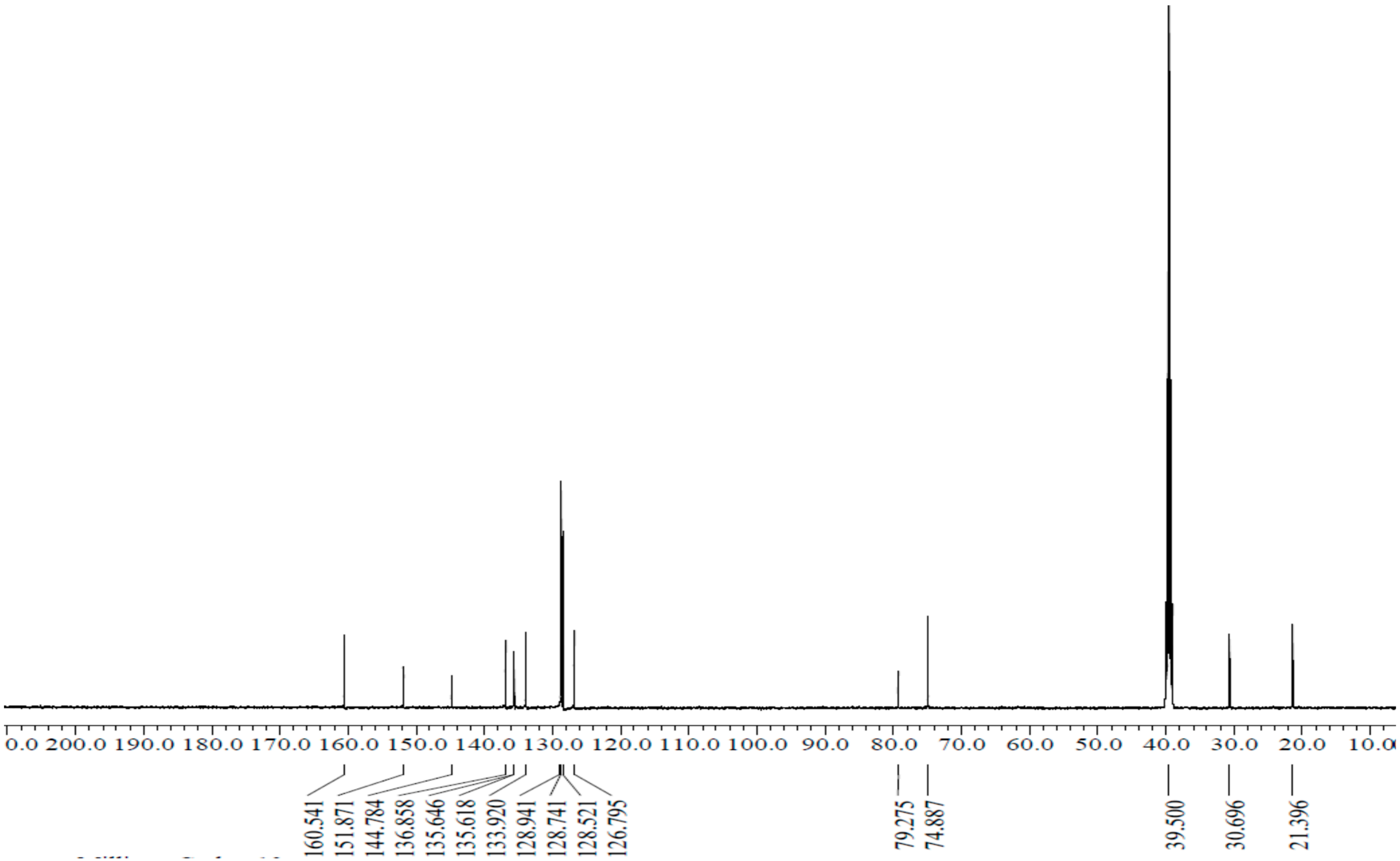

Figure S11: ^{13}**C NMR Spectrum of compound 5a**

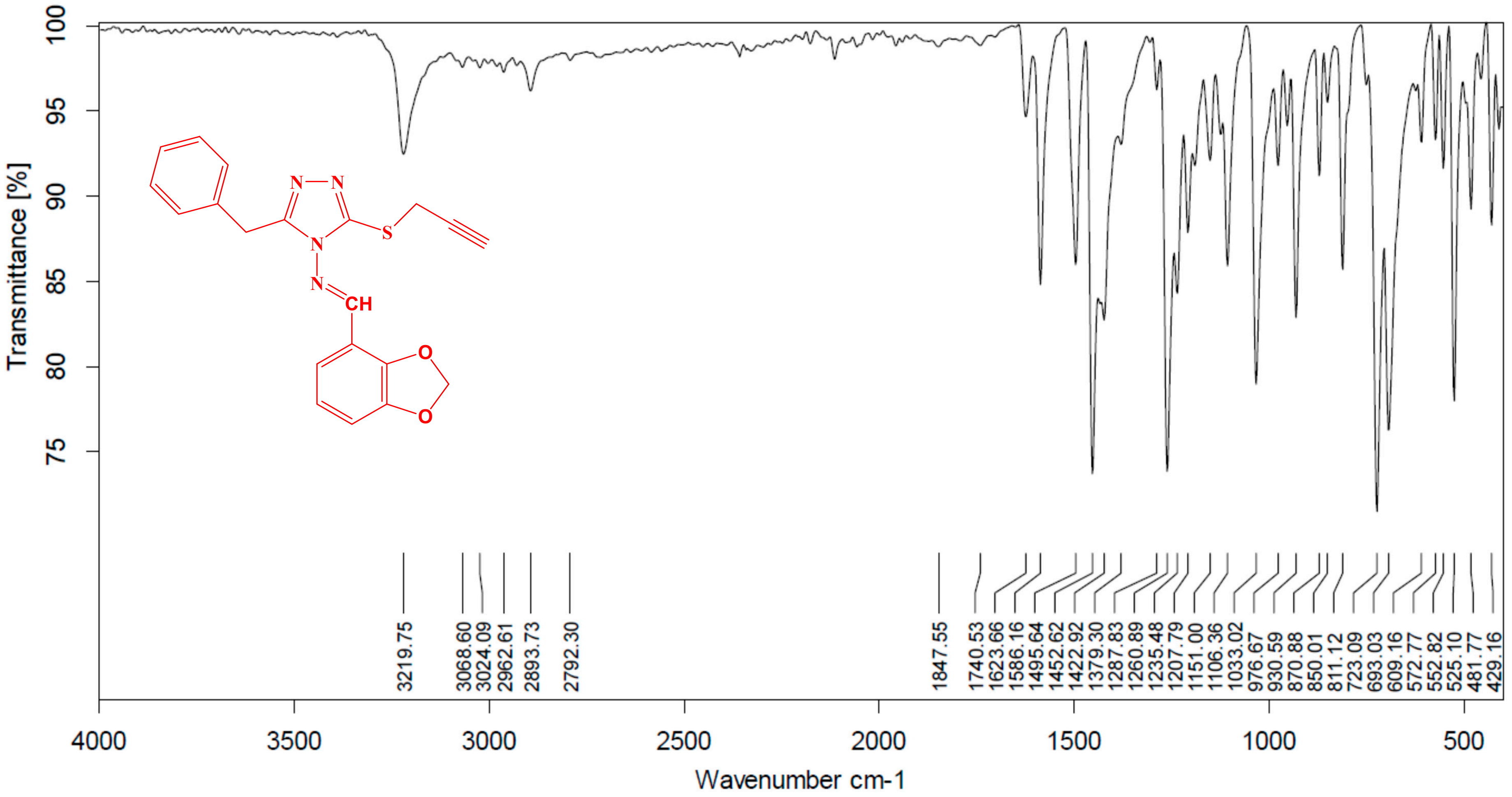

Figure S12: IR Spectrum of compound 5b

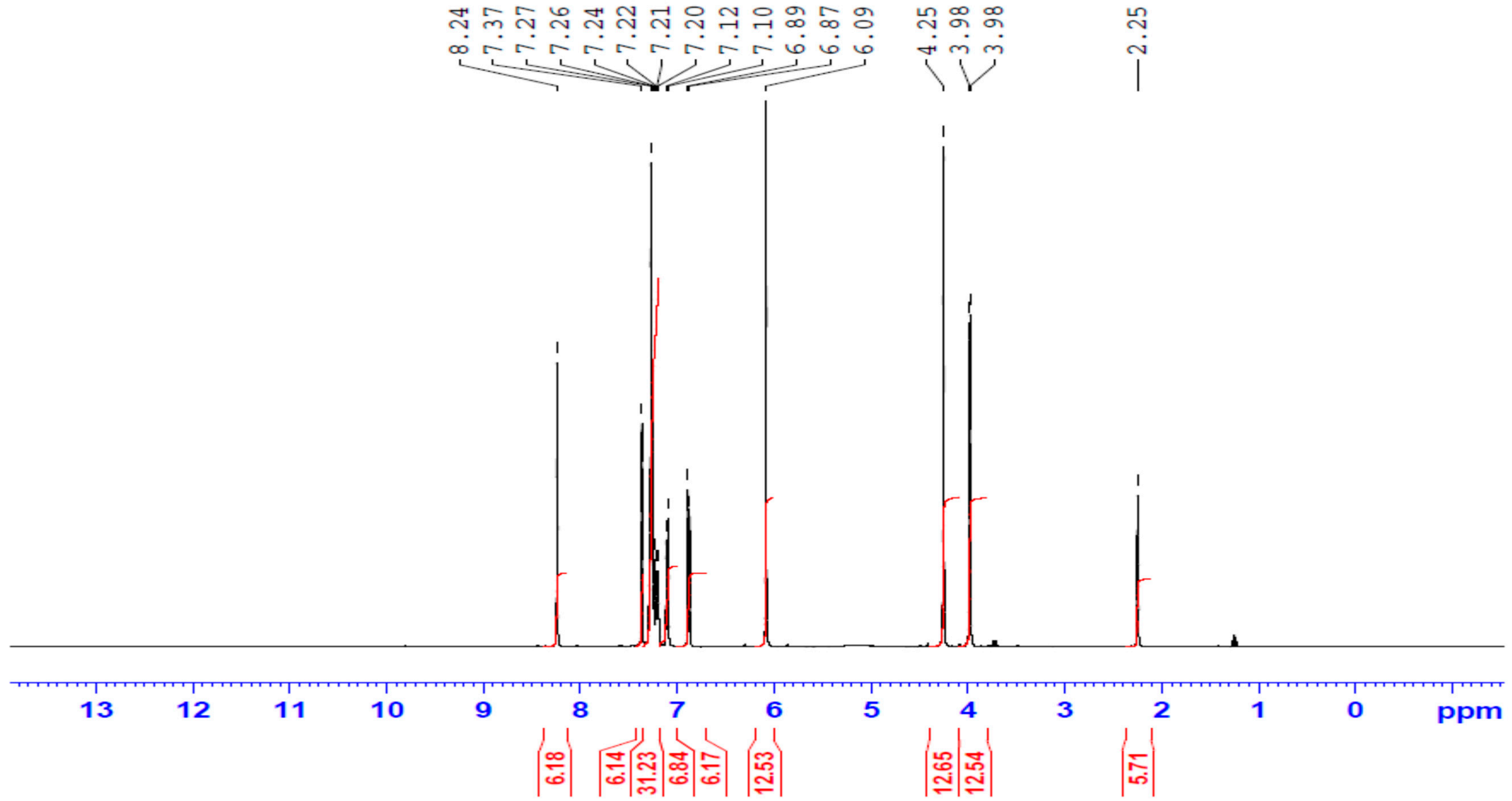

Figure S13: ^{1}H NMR Spectrum of compound 5b

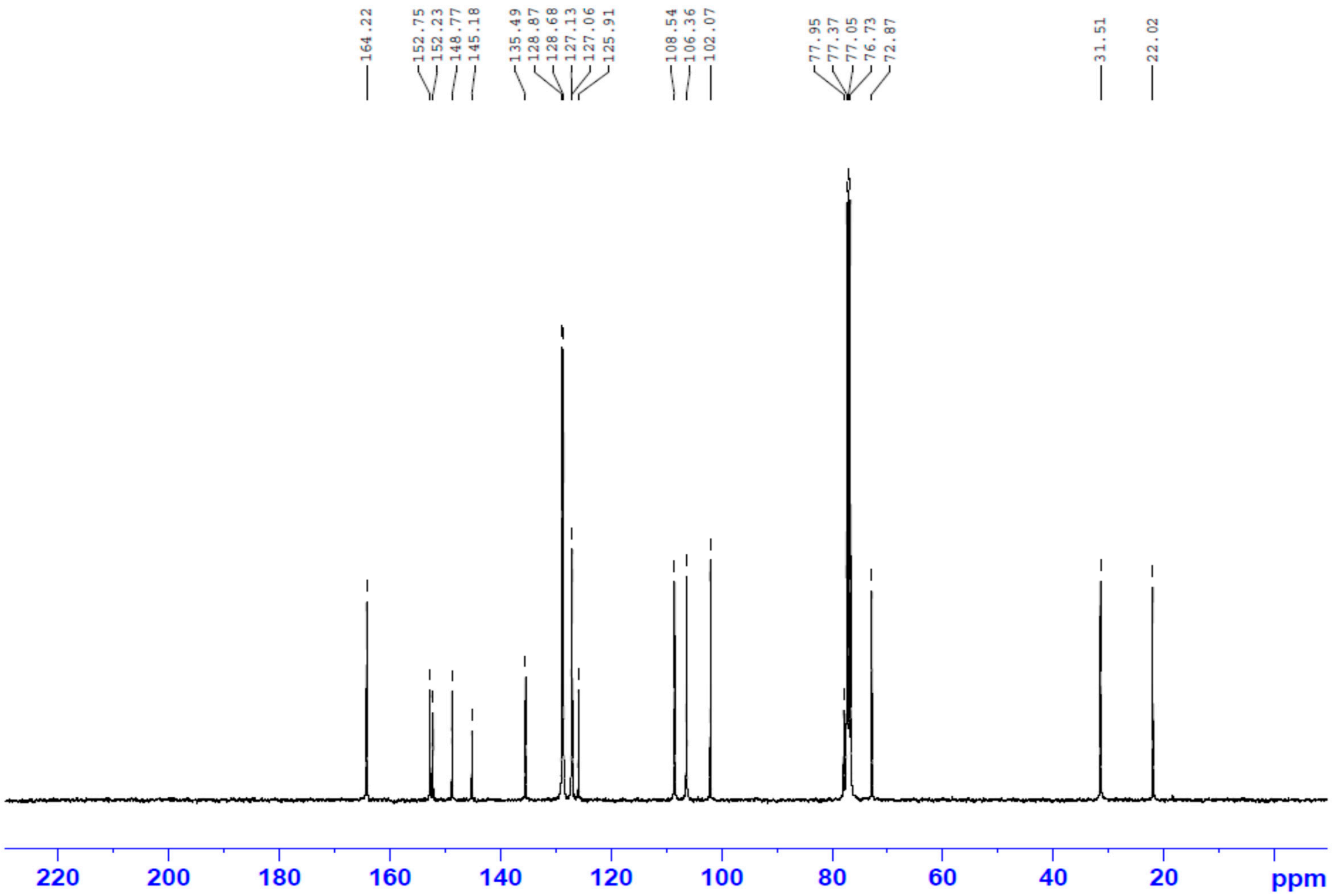

Figure S14: ^{13}**C NMR Spectrum of compound 5b**

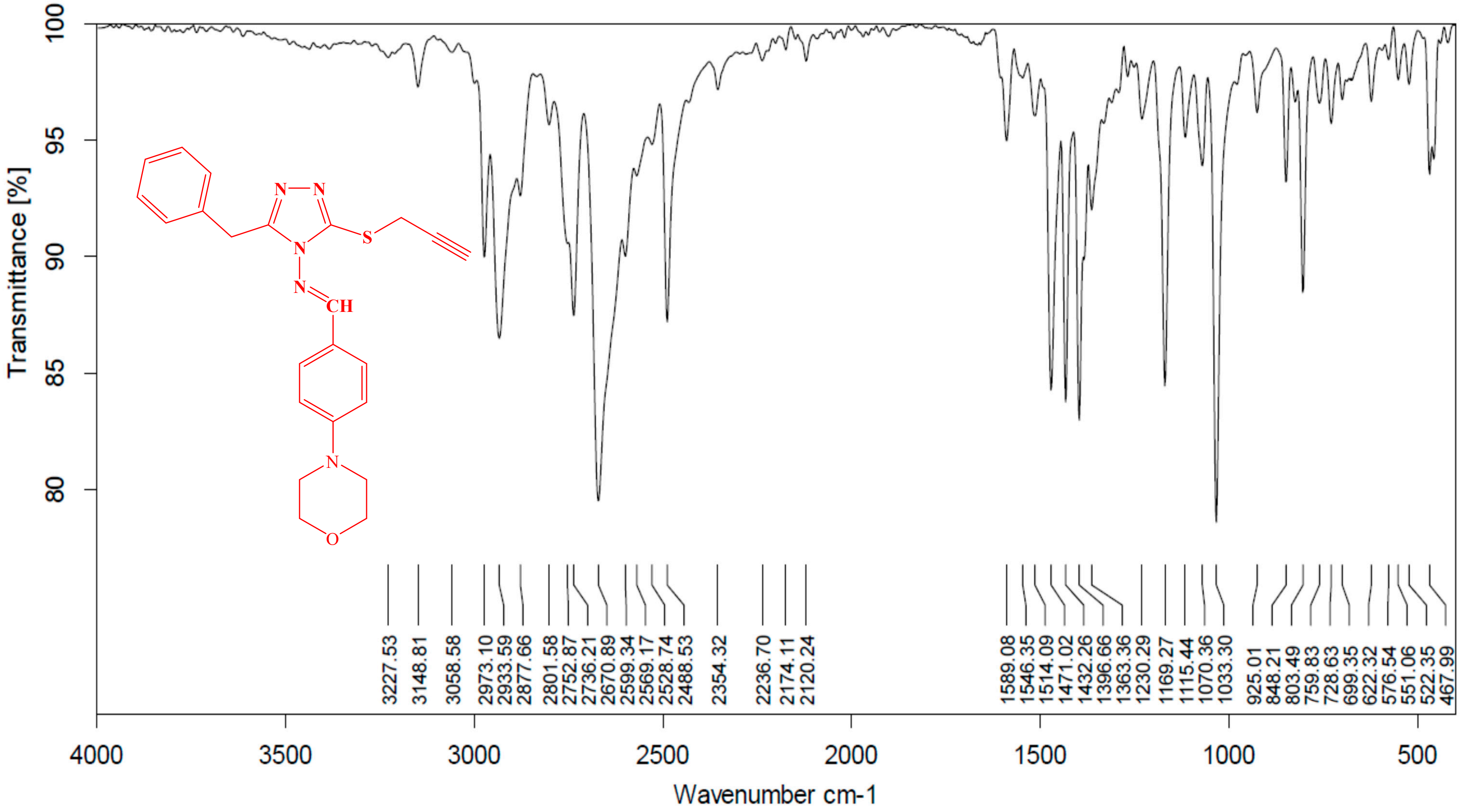

Figure S15: IR Spectrum of compound 5c

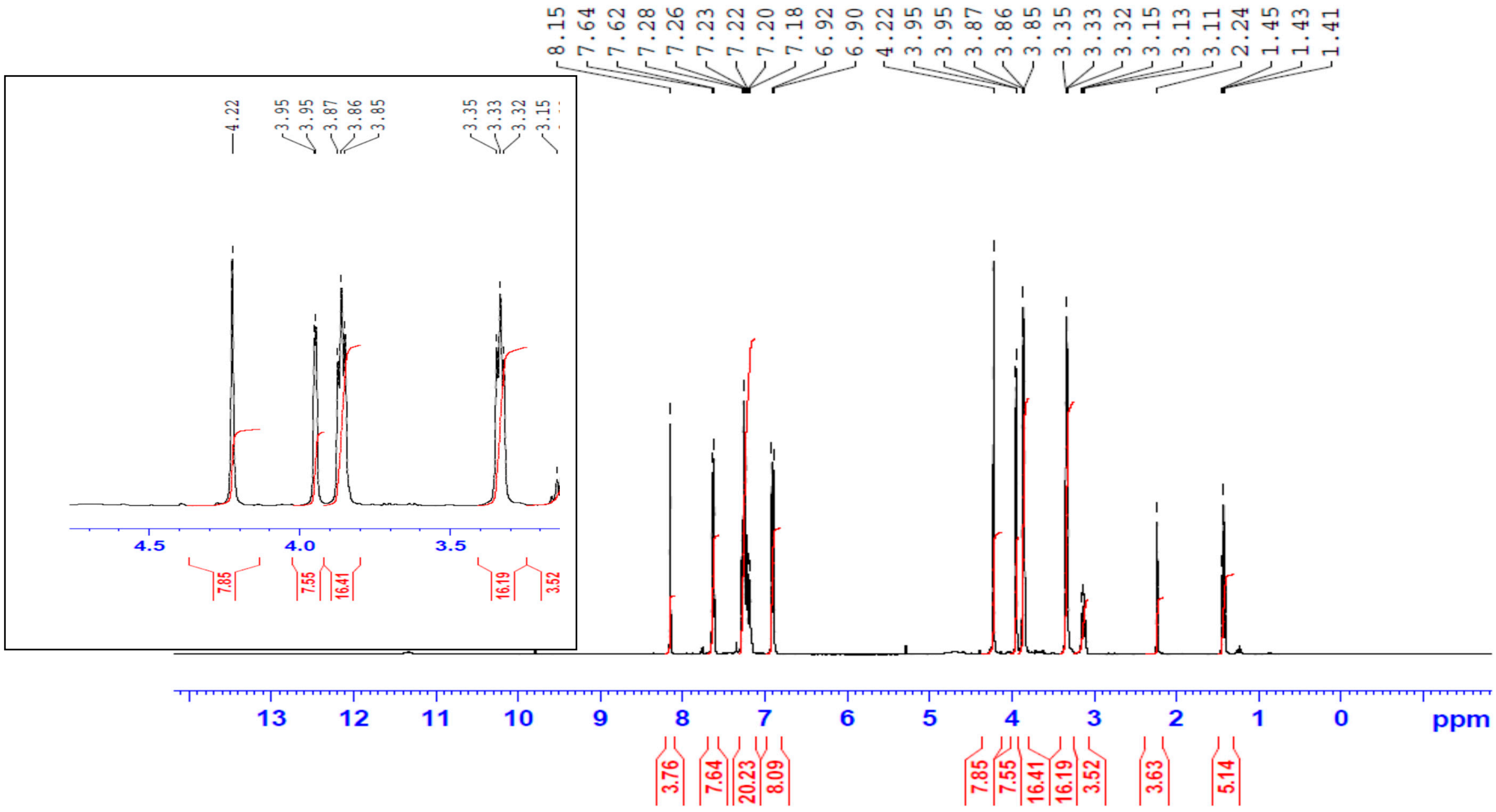

Figure S16: ^{1}H NMR Spectrum of compound 5c

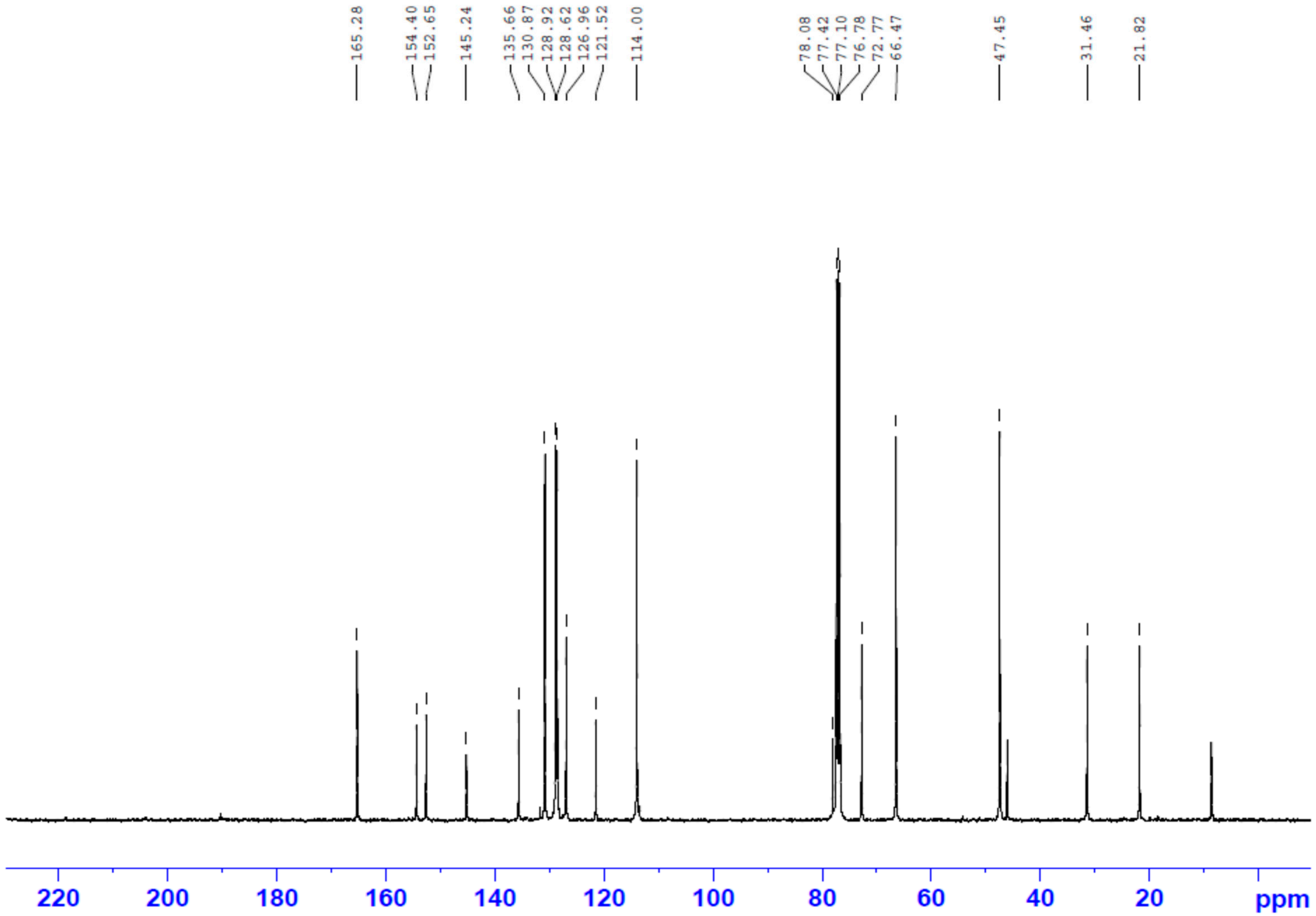

Figure S17: ^{13}C NMR Spectrum of compound 5c

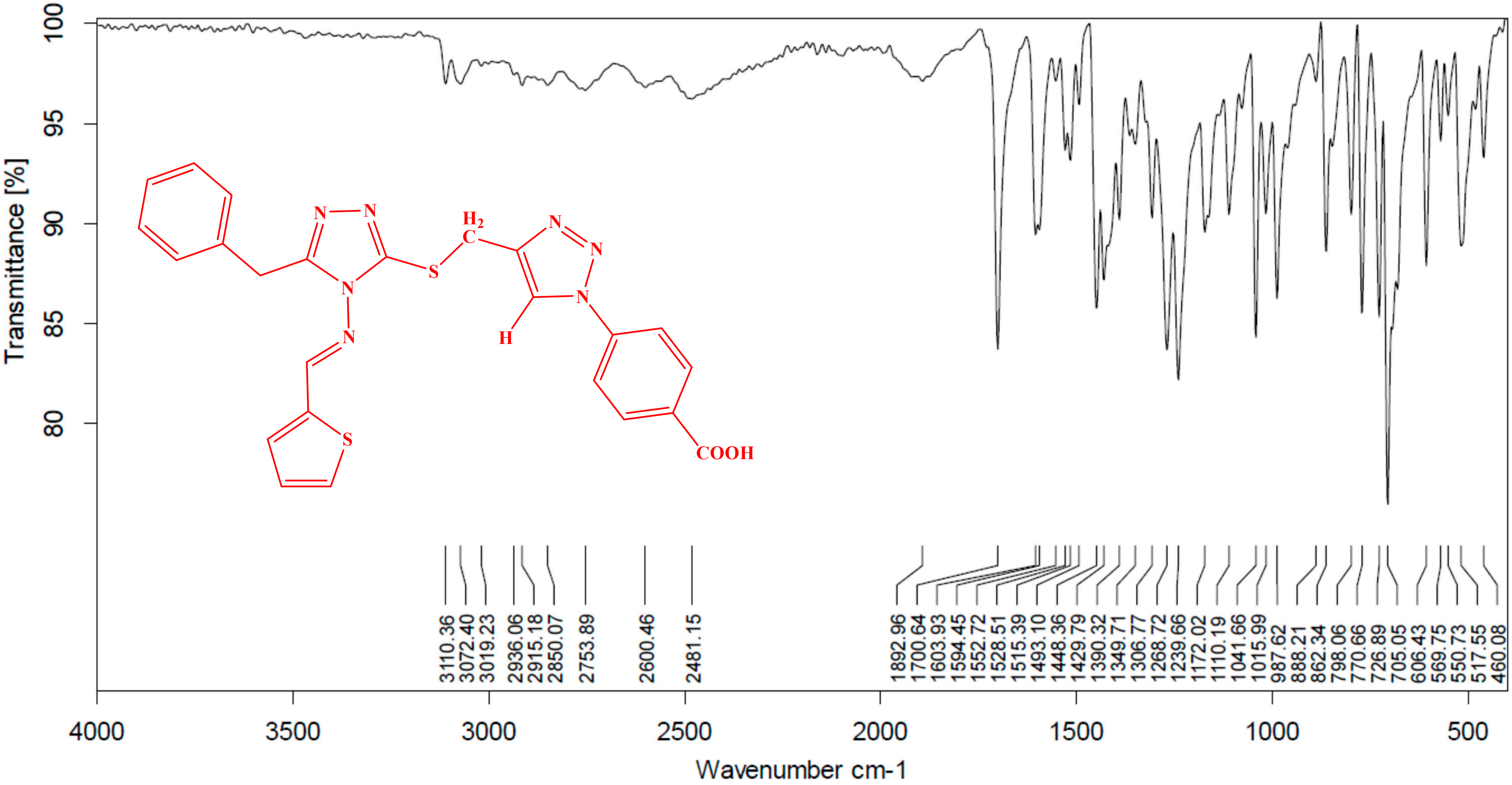

Figure S18: IR Spectrum of compound 7a

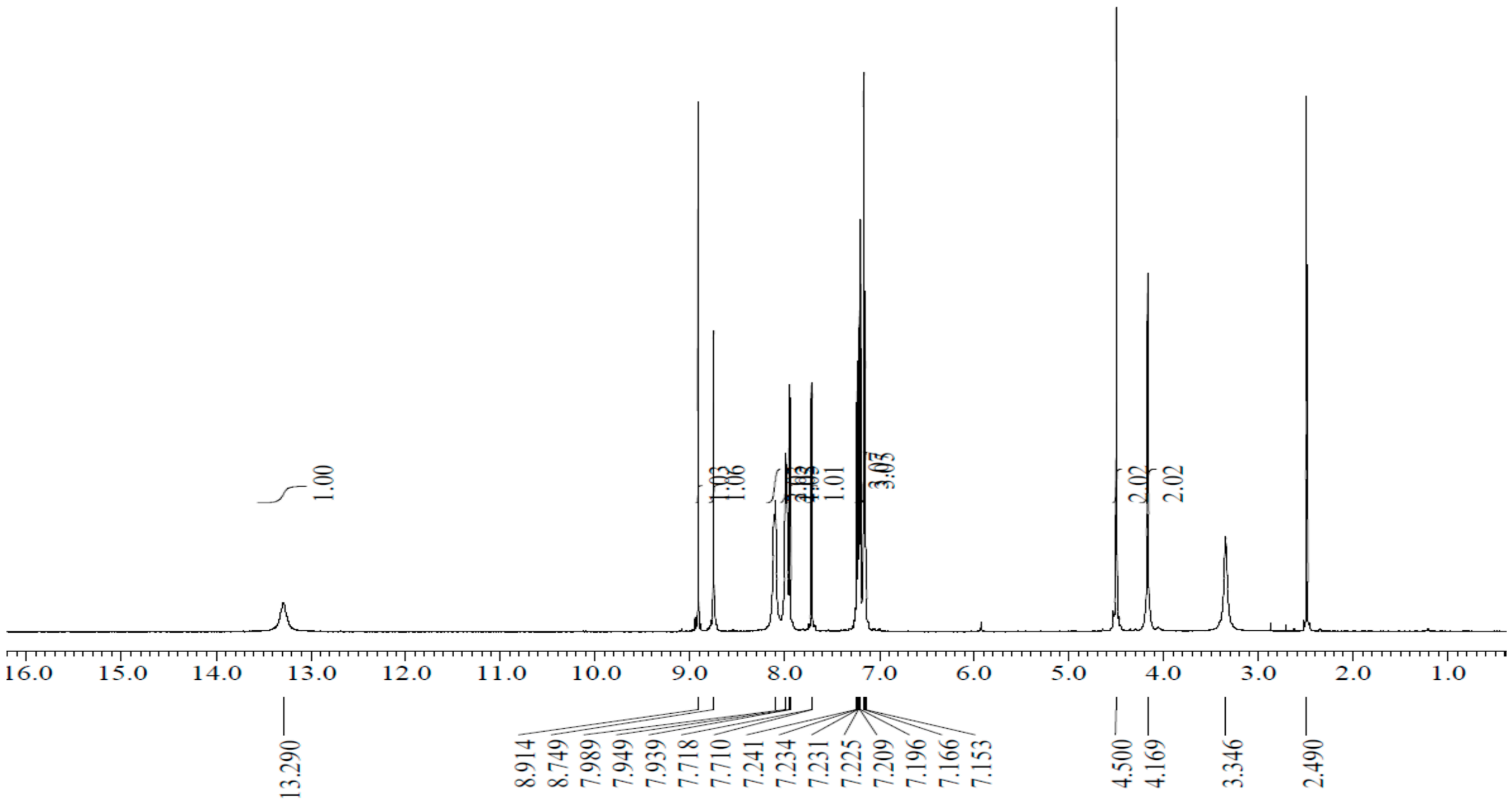

Figure S19: ^{1}H NMR Spectrum of compound 7a

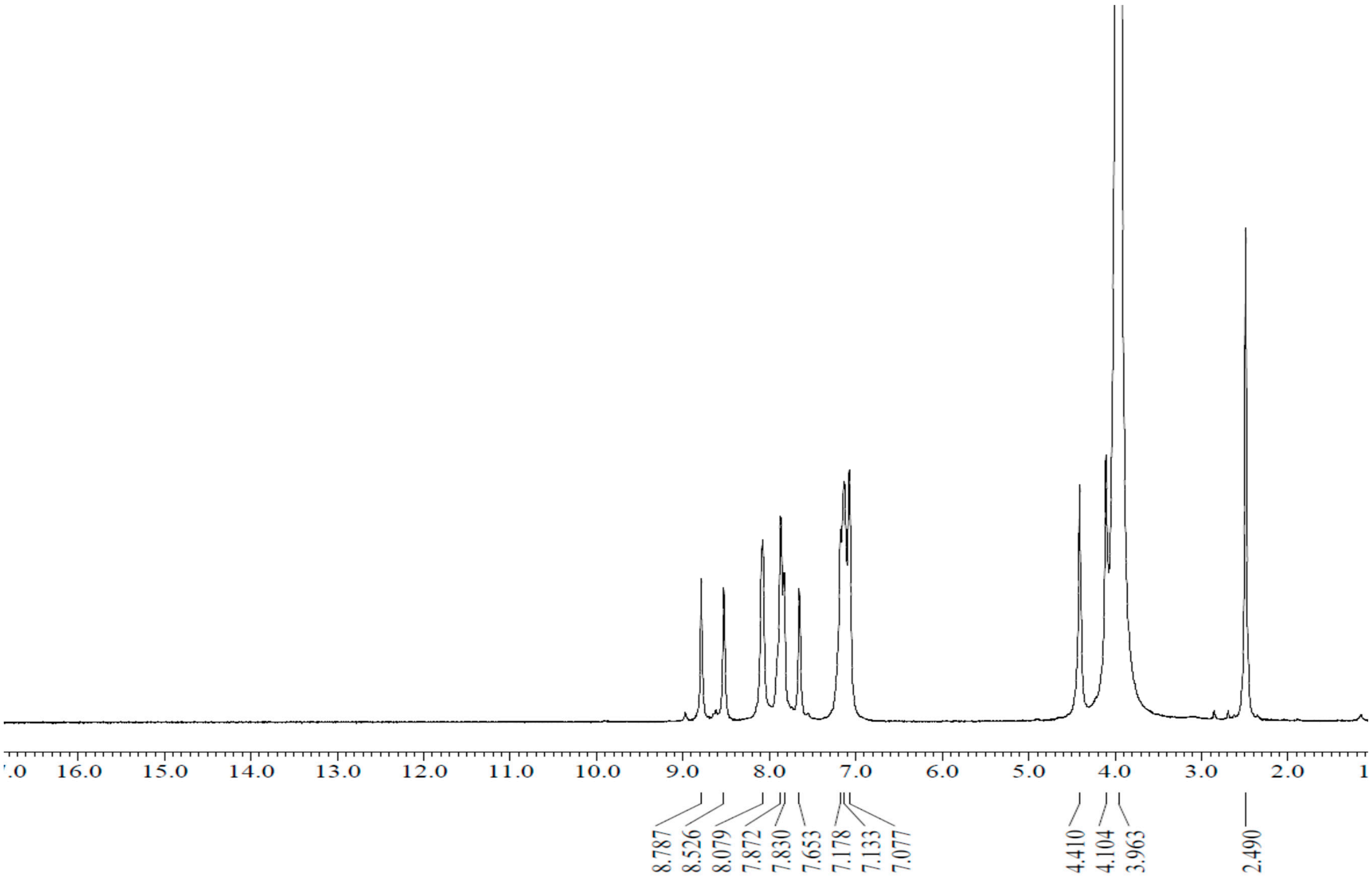

Figure S20: D_2O **Spectrum of compound 7a**

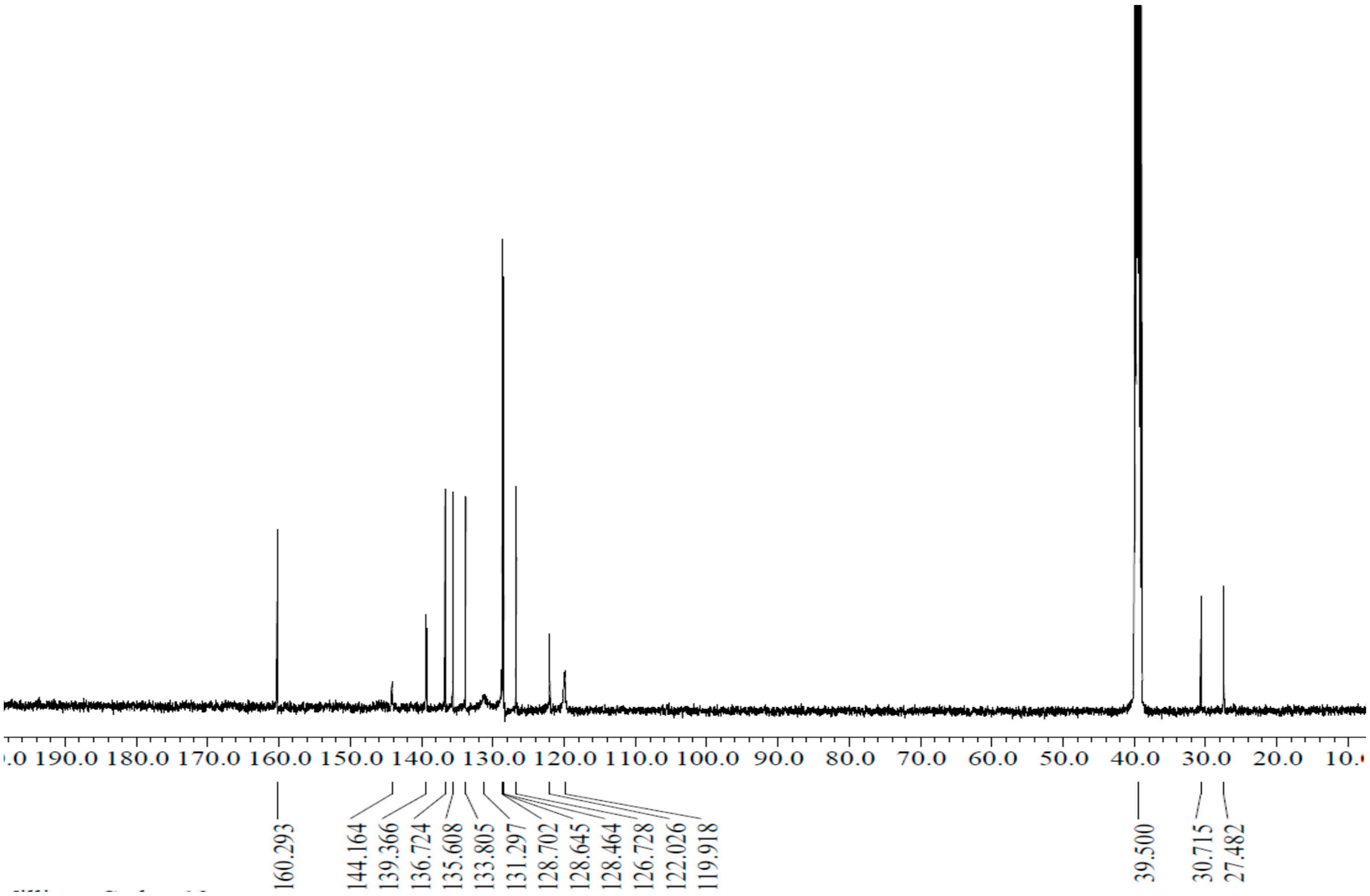

Figure S21: ^{13}**C NMR Spectrum of compound 7a**

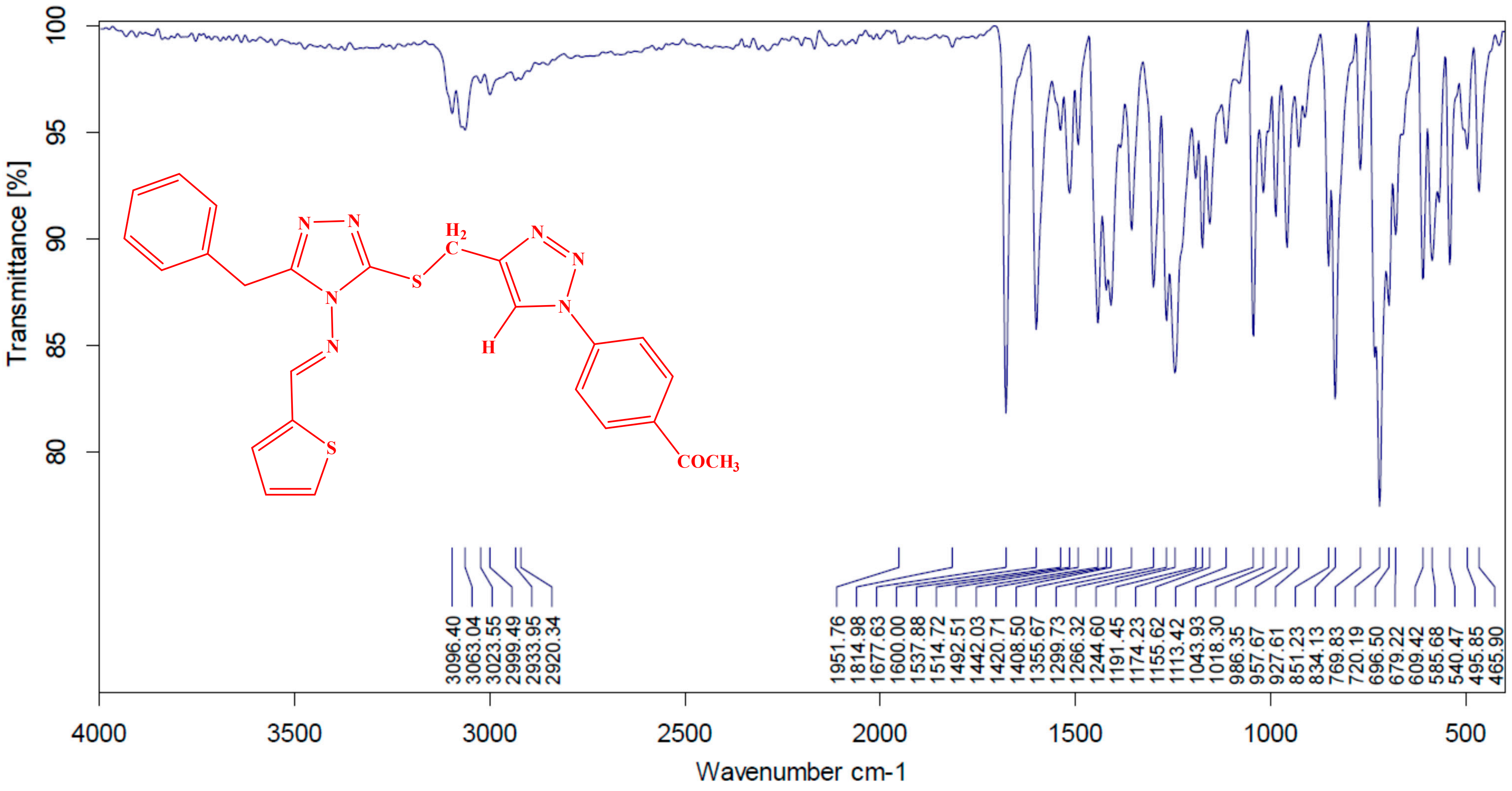

Figure S22: IR Spectrum of compound 7b

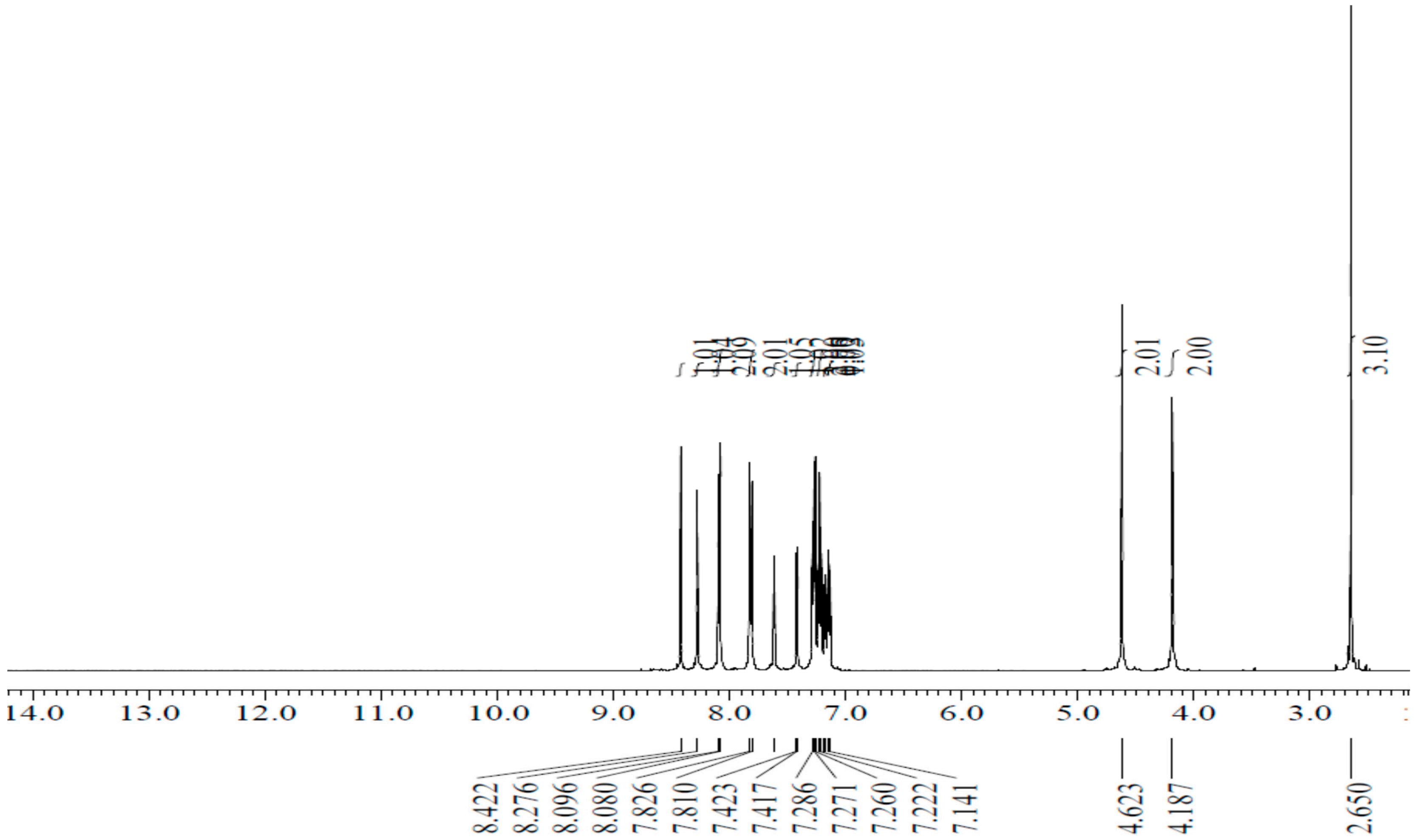

Figure S23: ^{1}H NMR Spectrum of compound 7b

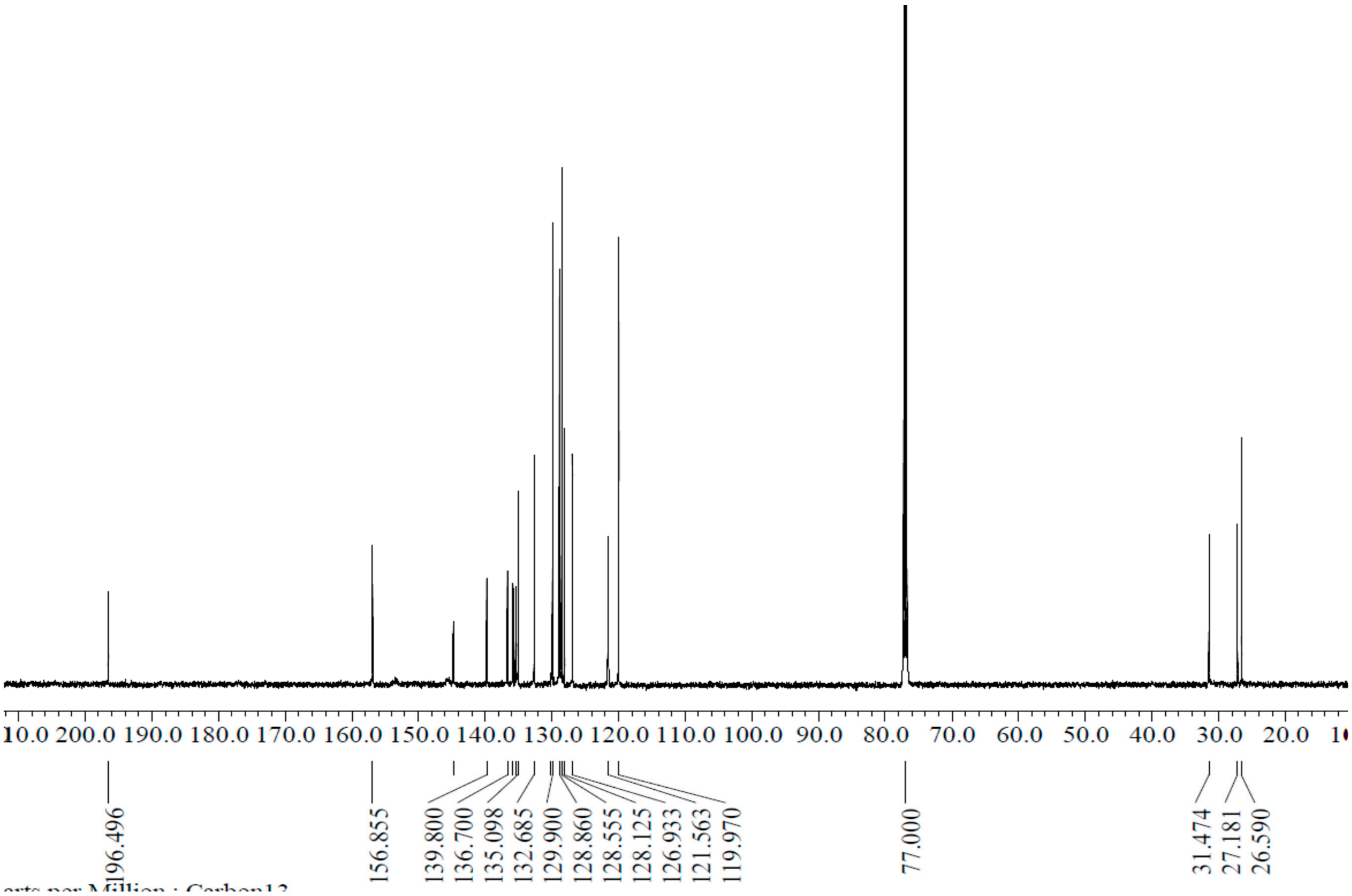

Figure S24: ^{13}C NMR Spectrum of compound 7b

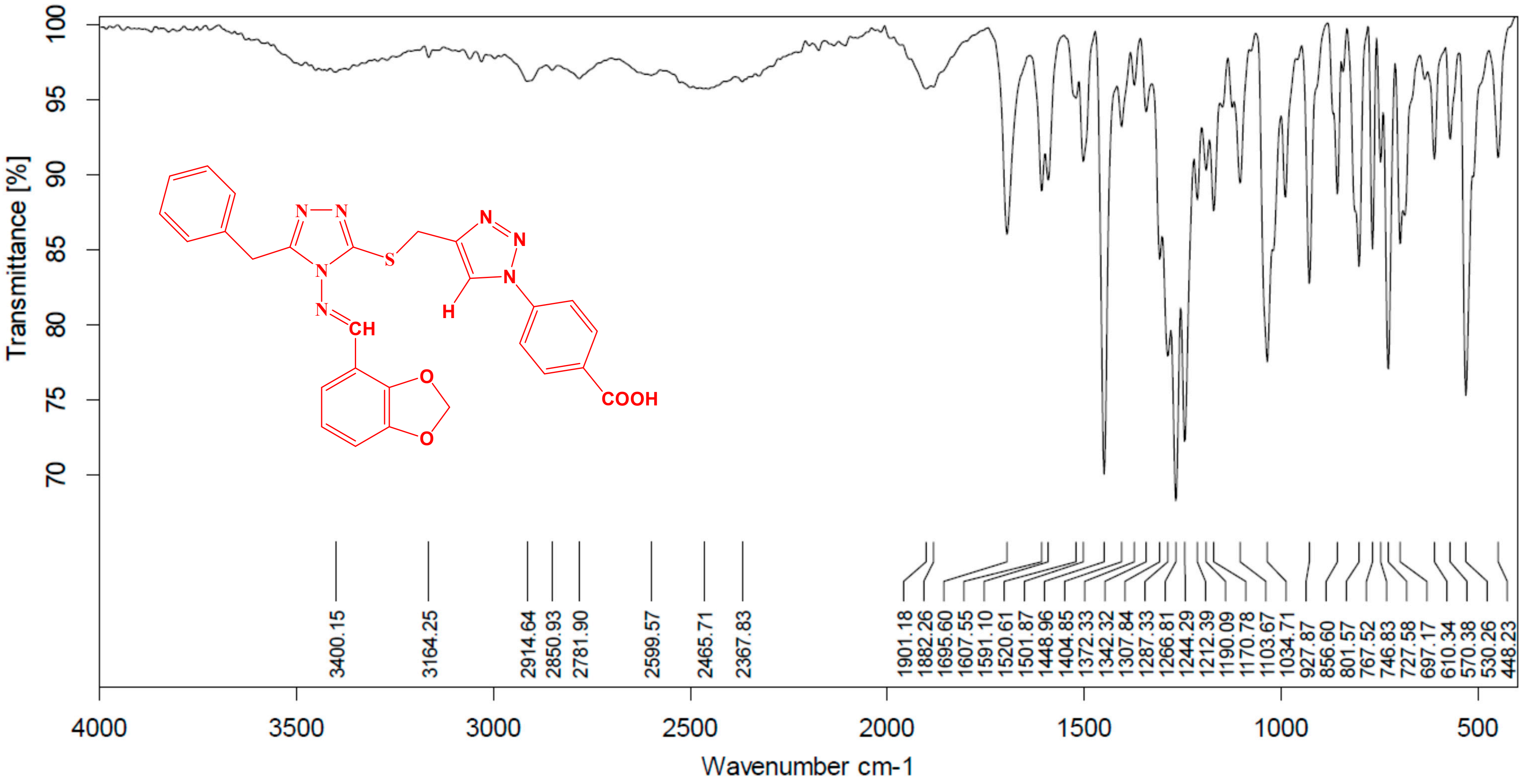

Figure S25: IR Spectrum of compound 7c

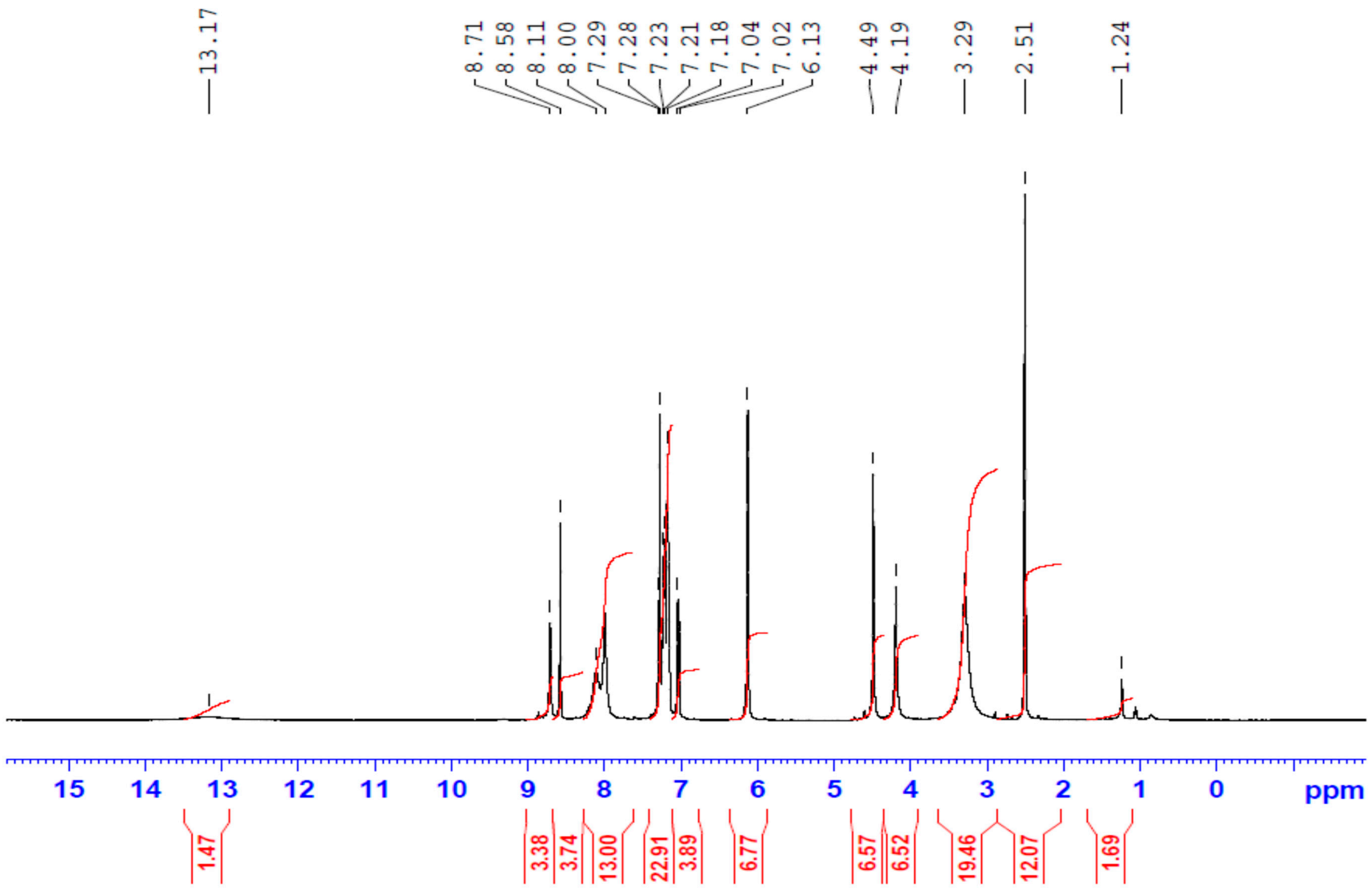

Figure S26: ^{1}H NMR Spectrum of compound 7c

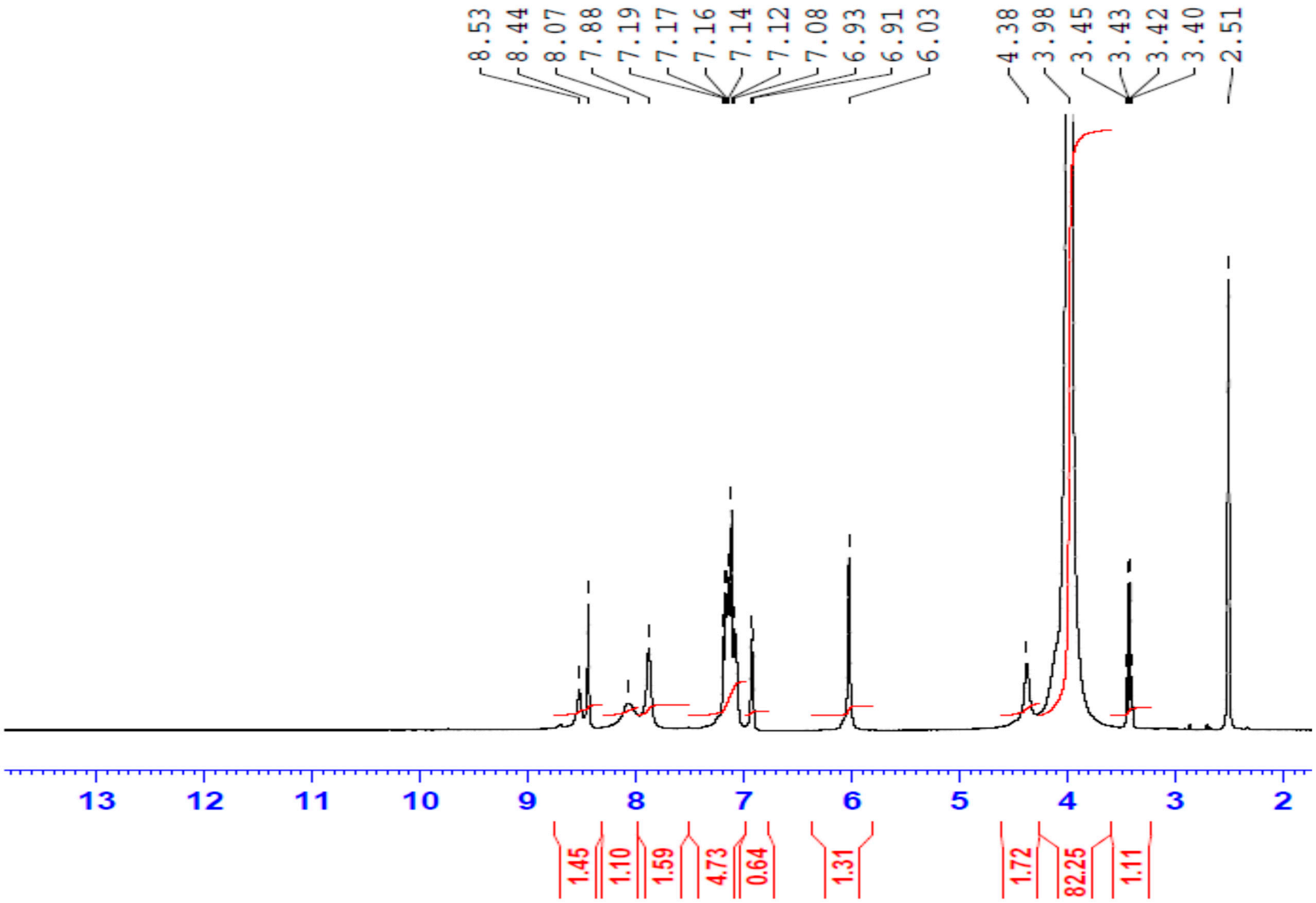

Figure S27: D_2O Spectrum of compound 7c

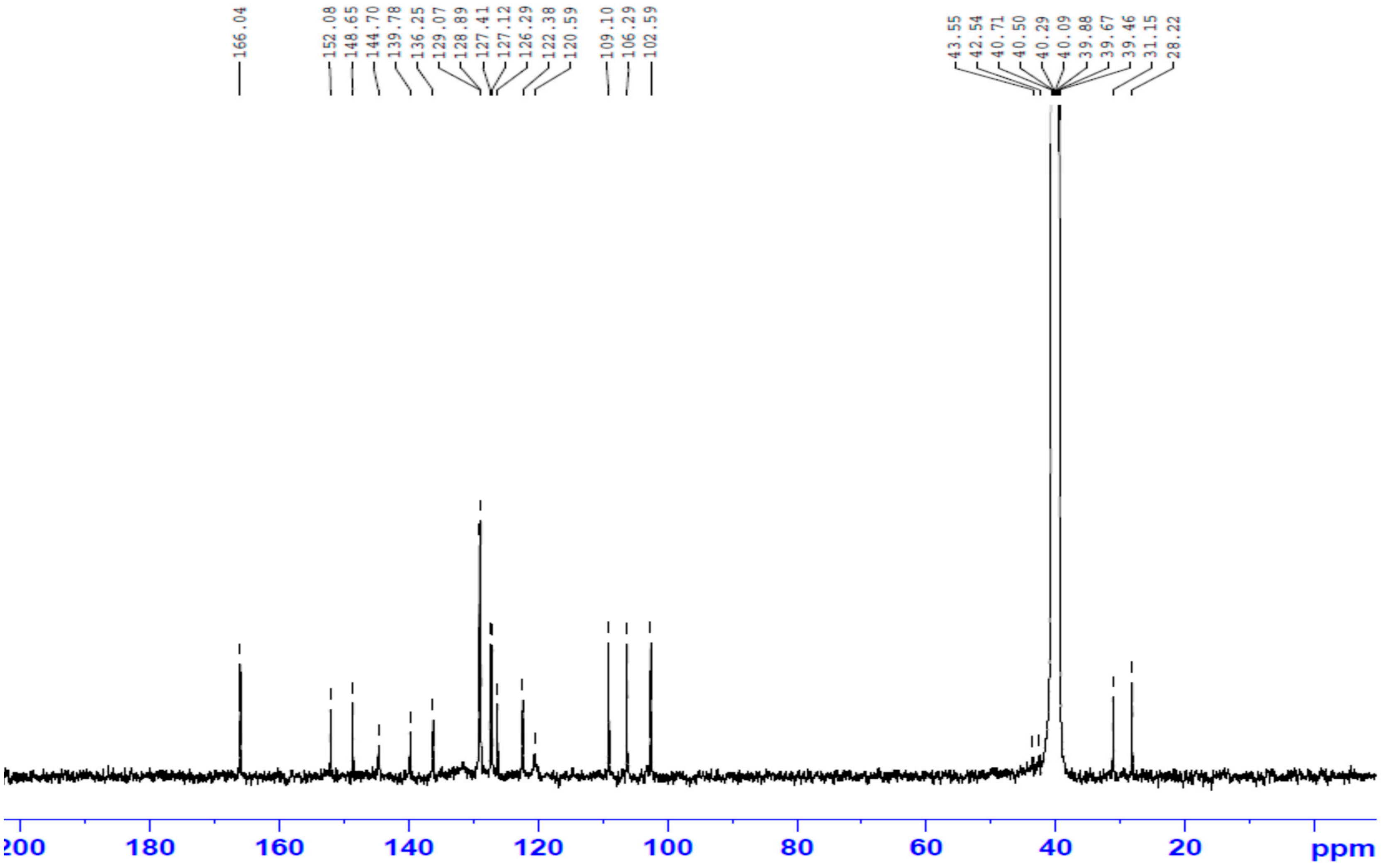

Figure S28: ^{13}**C NMR Spectrum of compound 7c**

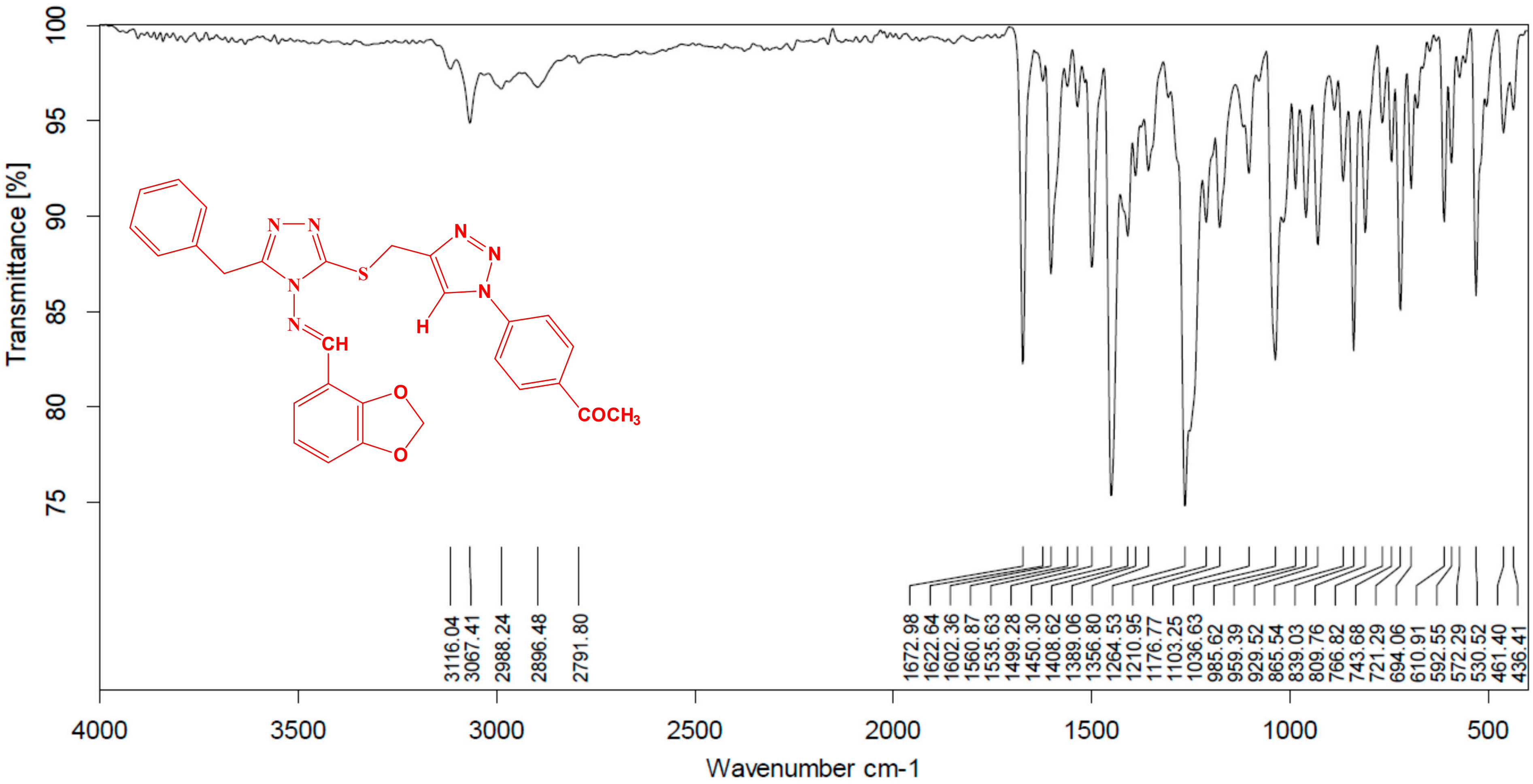

Figure S29: IR Spectrum of compound 7d

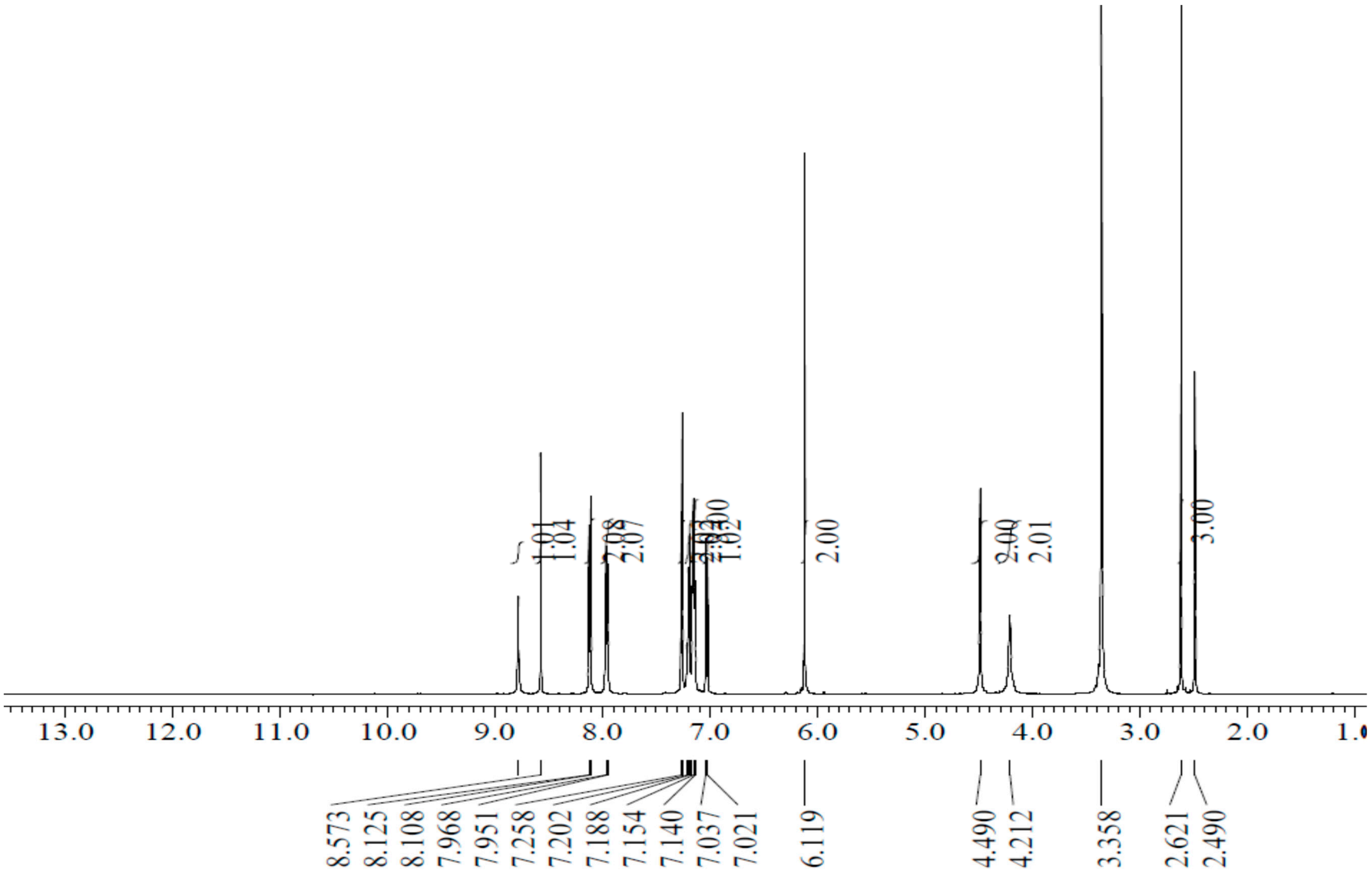

Figure S30: ^{1}**H NMR Spectrum of compound 7d**

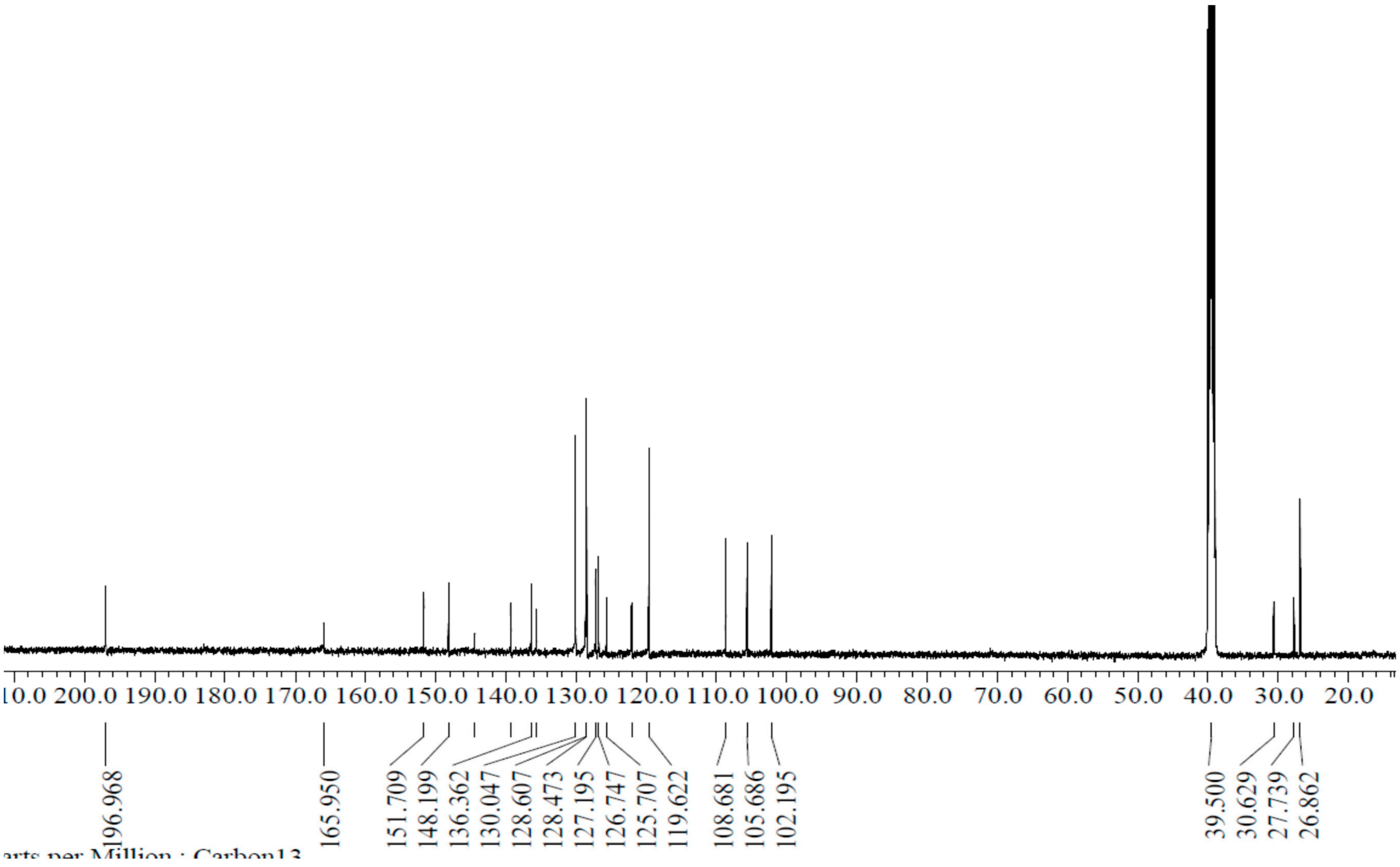

Figure S31: ^{13}C NMR Spectrum of compound 7d

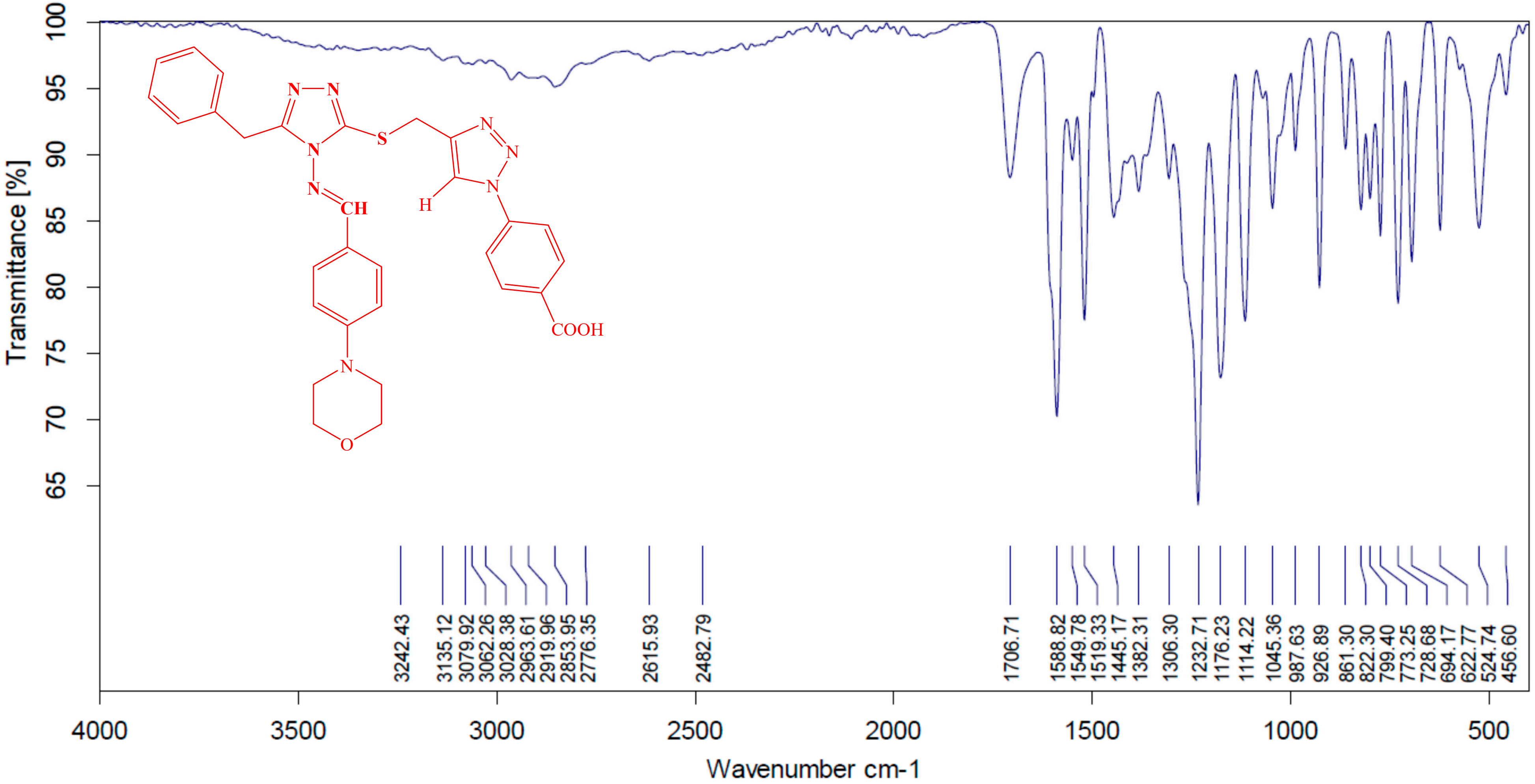

Figure S32: IR Spectrum of compound 7e

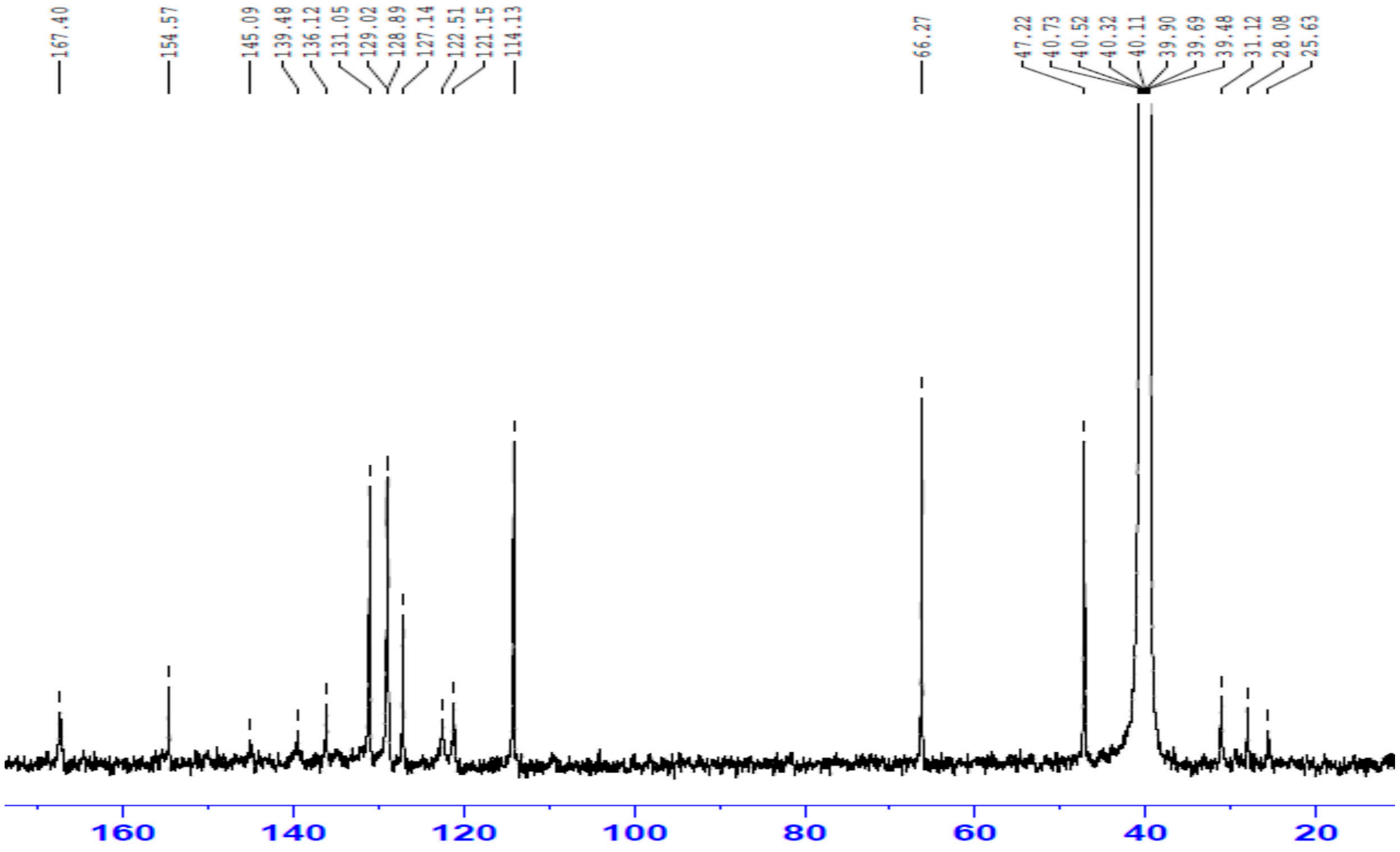

Figure S33: ^{13}**C NMR Spectrum of compound 7e**

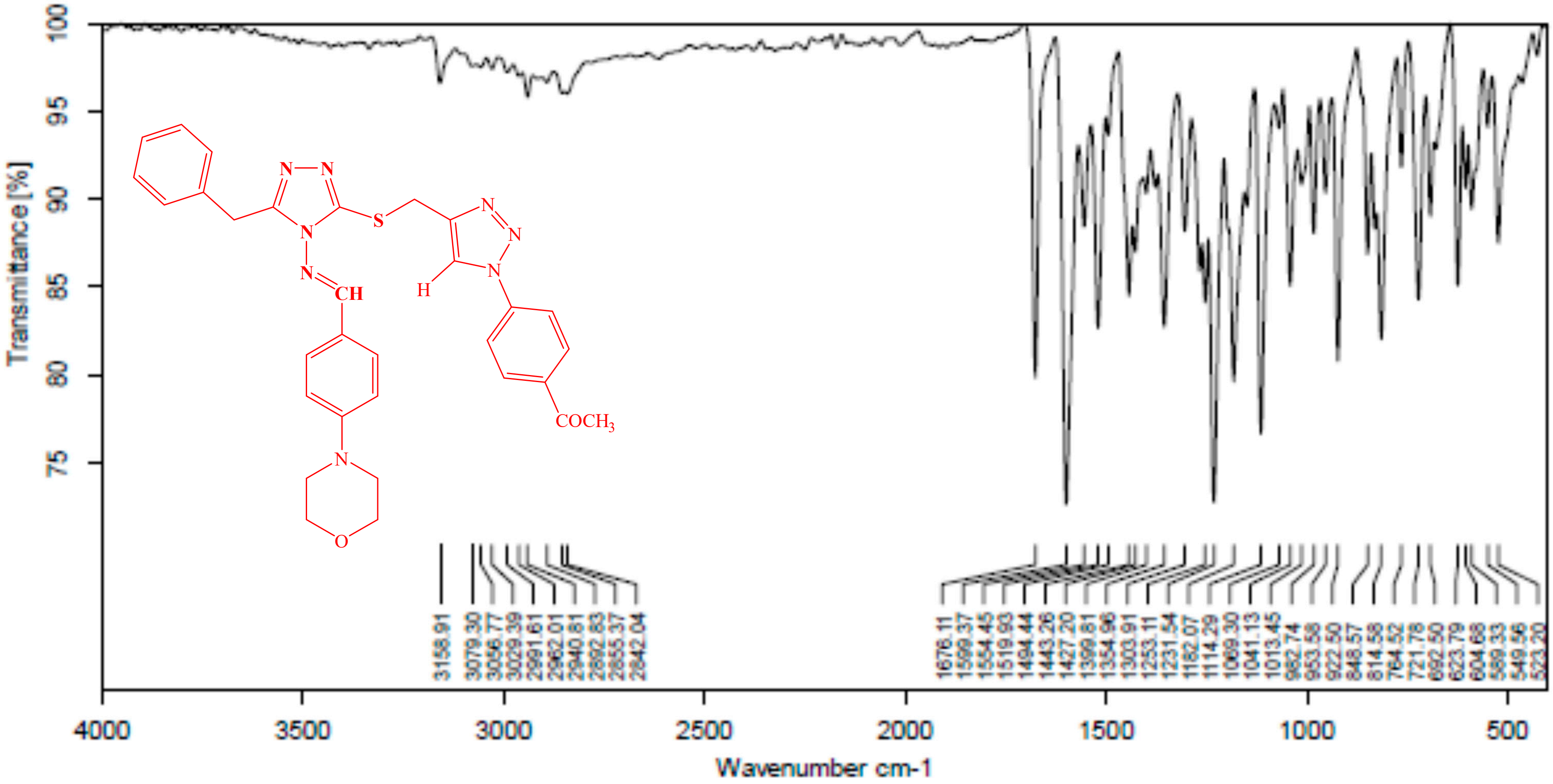

Figure S34: IR Spectrum of compound 7f

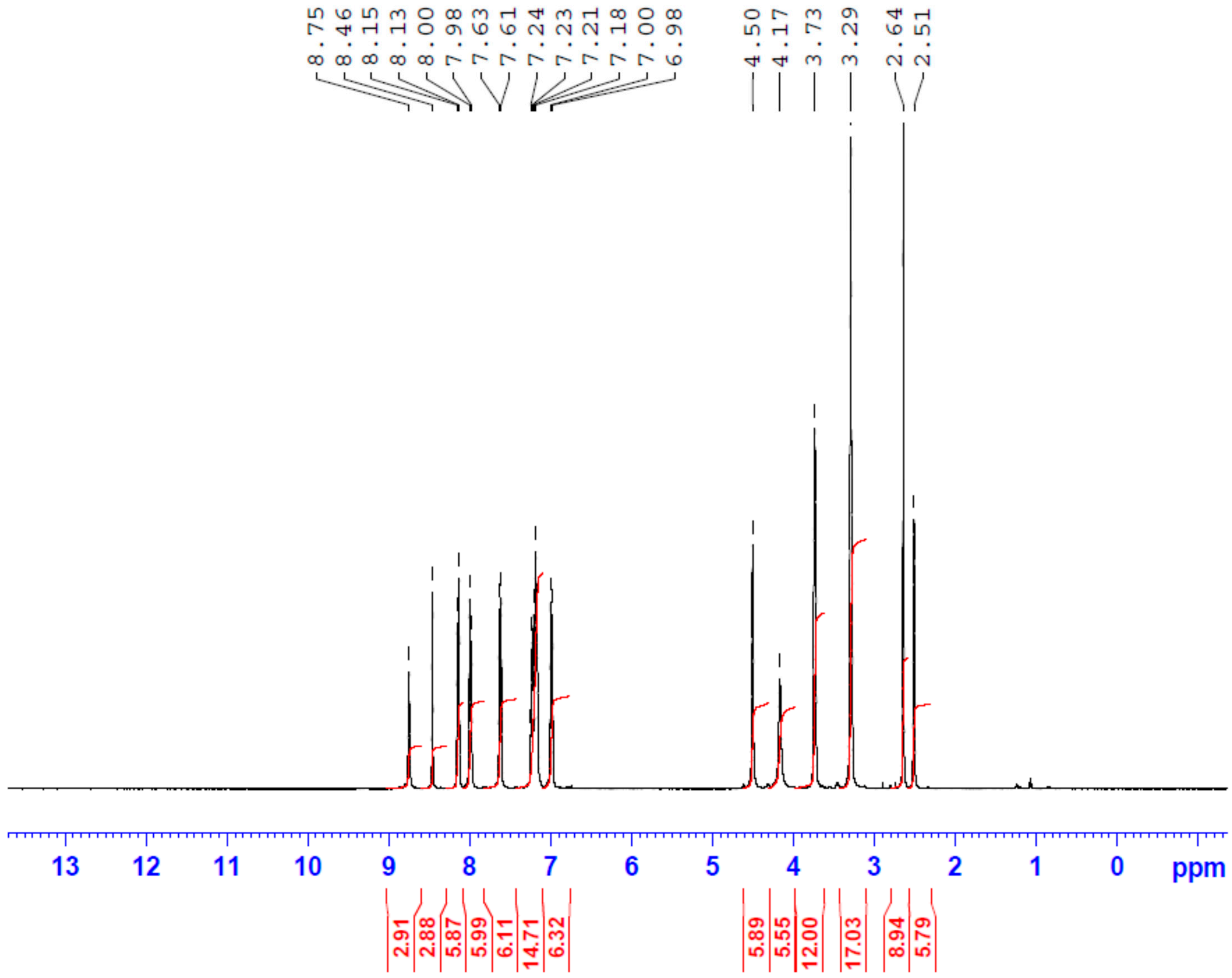

Figure S35: ^{1}**H NMR Spectrum of compound 7f**

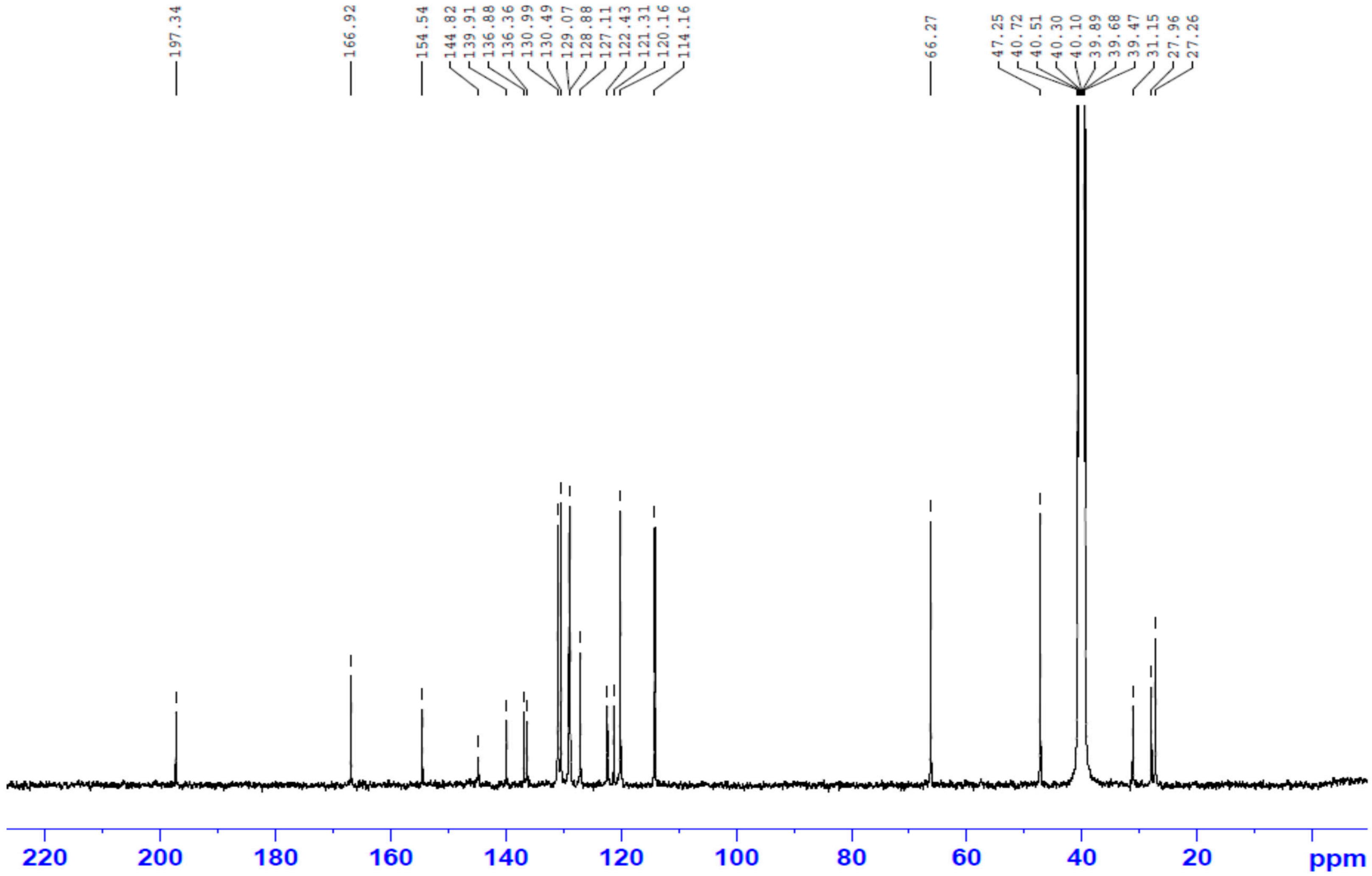

Figure S36: ^{13}**C NMR Spectrum of compound 7f**
